# A Novel PROTAC Confers a Dual Benefit Against Amyloid and Tau Pathology in Alzheimer's Disease via DAPK1 Degradation

**DOI:** 10.7150/ijbs.131465

**Published:** 2026-04-23

**Authors:** Ruomeng Li, Jing Yao, Wen Peng, Lizhen Zheng, Xueyin Wu, Xindong Shui, Xiaoqing Zheng, Wujin Tian, Long Wang, Ying Zhou, Xinglin Ruan, Xiaodong Pan, Tao Zhang, Yang Liu, Tae Ho Lee, Dongmei Chen

**Affiliations:** 1 Fujian Key Laboratory of Cognitive Function and Diseases, Institute of Basic Medicine, School of Basic Medical Sciences, Fujian Medical University, Fuzhou, Fujian, China.; 2 Department of Medicinal Chemistry, School of Pharmacy, Fujian Medical University, Fuzhou, Fujian, China.; 3 Fujian Key Laboratory of Natural Medicine Pharmacology, School of Pharmacy, Fujian Medical University, Fuzhou, Fujian, China.; 4 Neurological Medical Center, Fujian Medical University Union Hospital, Fuzhou, Fujian, China.; 5 Department of Neurology, Center for Cognitive Neurology, Fujian Medical University Union Hospital, Fuzhou, Fujian, China.

**Keywords:** Alzheimer's disease (AD), death-associated protein kinase 1 (DAPK1), proteolysis-targeting chimera (PROTAC), tau phosphorylation, amyloid-β (Aβ)

## Abstract

Alzheimer's disease (AD) is a neurodegenerative disorder that is caused by multiple factors, characterized by a progressive decline in cognitive ability, extracellular amyloid-β (Aβ) plaques, and intracellular neurofibrillary tangles composed of hyperphosphorylated tau. Current treatment strategies can provide only symptomatic treatment or limited efficacy, highlighting the need to intervene in the upstream regulatory factors that drive both amyloid and tau pathologies. Death-associated protein kinase 1 (DAPK1) is a key driver upstream of both amyloid precursor protein processing and tau phosphorylation, simultaneously promoting amyloidogenesis and tau-mediated pathology in AD. In this study, we developed CP1, a bifunctional proteolysis-targeting chimera (PROTAC), to recruit E3 ubiquitin ligase to DAPK1, thereby inducing the ubiquitination and proteasomal degradation of DAPK1. CP1 efficiently eliminated the DAPK1 protein in primary cortical neurons without affecting its mRNA level, resulting in reduced Aβ generation and tau hyperphosphorylation. *In vivo*, upon systemic administration, CP1 effectively crossed the blood-brain barrier, degraded DAPK1, and consequently reduced the Aβ plaque burden and mitigated neuroinflammation in female 5xFAD mice. In a AAV-hTau-P301L tauopathy model, CP1 treatment suppressed tau hyperphosphorylation, preserved NeuN- and MAP2-positive neurons, attenuated astrocytic and microglial activation, and ultimately restored learning and memory abilities in both male and female mice. In summary, these findings demonstrate that degrading DAPK1 via a PROTAC strategy simultaneously mitigates both amyloid and tau pathology, indicating that CP1 is an effective candidate for disease-modifying therapy.

## Introduction

Alzheimer's disease (AD) is a progressive neurodegenerative disorder and is the leading cause of dementia worldwide, affecting more than 50 million individuals 1. Clinically, AD presents as progressive memory loss and cognitive decline; pathologically, it is characterized by the accumulation of extracellular amyloid-β (Aβ) plaques and intracellular neurofibrillary tangles composed of hyperphosphorylated tau protein 2. Despite decades of research, existing treatments, including recently developed anti-amyloid antibodies, provide only modest symptomatic relief and do not halt disease progression 3, 4. Similarly, therapeutic approaches directly targeting tau have shown limited clinical efficacy, underscoring the need to identify upstream regulators that drive both amyloid and tau pathology and to develop approaches capable of their coordinated suppression 5-9.

Death-associated protein kinase 1 (DAPK1), a calcium/calmodulin-dependent serine/threonine kinase, has emerged as a critical node that regulates multiple neurodegenerative pathways 10. DAPK1 is known for its role in apoptosis and autophagy 11. In neurons, DAPK1 regulates key processes such as cytoskeletal stability, mitochondrial dynamics, and synaptic homeostasis 10. Elevated levels of DAPK1 expression and activity have been consistently observed in postmortem AD brains and in transgenic models, where they correlate with synaptic loss, tau hyperphosphorylation, and neuronal apoptosis 12. Mechanistically, DAPK1 phosphorylates amyloid precursor protein (APP) at Thr668 and tau at several AD-associated residues, including Thr231, Ser262, and Ser396 10. These phosphorylation events promote amyloidogenic APP processing and facilitate tau misfolding, thereby concurrently amplifying two core pathogenic pathways in AD 13. Moreover, we recently reported that DAPK1 phosphorylates and degrades SUMO specific peptidase 1 (SENP1), thereby increasing tau SUMOylation and contributing to the aggravation of tau pathology and associated cognitive impairment 14. Furthermore, dysregulated DAPK1 signaling contributes to calcium overload, mitochondrial depolarization, and caspase activation, establishing its role as a central driver of neuronal death in AD 15. Given its convergent influence on both amyloid and tau pathology, DAPK1 represents a promising therapeutic target for disease modification 16. Previous studies from our group and others have demonstrated that genetic silencing or pharmacological inhibition of DAPK1, such as by small-molecule inhibitors, effectively reduces tau phosphorylation, alleviates synaptic deficits, and mitigates Aβ deposition in both cellular and animal models 17-24. However, most conventional small-molecule inhibitors target the ATP-binding site and often exhibit poor kinase selectivity 25. They also show an inability to degrade the DAPK1 protein, leading to rapid recovery of kinase activity upon drug clearance. These limitations highlight the need for therapeutic strategies that achieve sustained suppression through target removal rather than transient inhibition.

Targeted protein degradation (TPD) via proteolysis-targeting chimeras (PROTACs) is a promising alternative therapeutic strategy 26. PROTAC technology involves the use of bifunctional molecules that recruit E3 ubiquitin ligases to specific target proteins, thereby inducing their ubiquitination and subsequent degradation by the proteasome 27. In contrast to occupancy-based inhibition, PROTAC-mediated degradation operates through a catalytic mechanism, offering the advantages of durability and often high specificity 28. Wang et al. developed a tau-targeting PROTAC and reported that it effectively reduced tau levels, thereby mitigating Aβ-induced neurotoxicity and improving synaptic and cognitive functions without apparent adverse effects 29. Studies have reported that PROTACs that degrade tau-related kinases can lower tau hyperphosphorylation and rescue cognitive deficits, supporting their therapeutic potential for treating tau-driven diseases 30, 31. These findings underscore the therapeutic promise of degrading tau via PROTACs for treating tauopathies and AD. However, whether PROTACs can similarly reduce Aβ deposition has not been established. We developed a DAPK1-directed PROTAC that conjugates a DAPK1-binding ligand to an E3 ligase recruiter via a flexible linker 32. Although its molecular design and degradation activity have been validated in our prior studies, its functional efficacy in relevant models remains to be established.

In addition, we aimed to address a clinically significant yet often overlooked aspect of AD pathogenesis: sex differences in disease susceptibility and progression. Epidemiological evidence indicates that compared with men, women have a greater incidence of AD, exhibit greater tau pathology, and experience more rapid cognitive decline 33. Hormonal depletion, particularly the loss of estrogen after menopause, along with sex-specific transcriptional and signaling profiles, may increase vulnerability to tau phosphorylation and synaptic degeneration 34. Preclinical studies likewise demonstrate that compared with male animals, female animals display increased tau pathology and neuronal loss 35. Despite these observations, the majority of mechanistic investigations in AD have relied primarily on male models, limiting translational relevance to the predominant patient population.

In this study, we demonstrated that CP1 efficiently induced the ubiquitination and proteasomal degradation of DAPK1, resulting in decreased phosphorylation of tau and APP and the suppression of Aβ production* in vitro*. Moreover, we primarily used female AD models, including both 5xFAD transgenic and AAV-hTau-P301L mice, alongside parallel cohorts of males, to evaluate the therapeutic efficacy of CP1. We showed that the systemic administration of CP1 markedly reduced the amyloid burden, attenuated tau hyperphosphorylation, preserved neuronal morphology, and rescued learning and memory deficits without inducing hematological or organ toxicity. Collectively, these findings establish the TPD of DAPK1 via a PROTAC as a selective and potent strategy to simultaneously counteract core AD pathologies and identify CP1 as a promising candidate for disease-modifying therapy.

## Materials and Methods

### Chemicals and reagents

MG132 (proteasome inhibitor) and various inhibitor cocktails (protease and phosphatase) were purchased from MedChemExpress (MCE, Shanghai, China) and Topscience (Shanghai, China). Cytosine-β-D-arabinofuranoside was obtained from MilliporeSigma (St. Louis, MO, USA). Reagents for transfection (TurboFect) and RNA extraction (FreeZol) were acquired from Thermo Fisher Scientific (Waltham, MA, USA) and Vazyme (Nanjing, China). UElandy (Suzhou, China) provided the CCK-8 kit. Custom rAAVs, including AAV-P301L (human mutant full-length Tau) and AAV-eGFP control, were produced by BrainVTA (Wuhan, China) 14.

### Cell culture

The human embryonic kidney 293T (HEK293T) cell line was obtained from the Cell Bank/Stem Cell Core Facility (Shanghai, China). The human neuroblastoma SH-SY5Y cell line was purchased from Procell Life Science & Technology (Wuhan, China). The SH-SY5Y cell line stably overexpressing human wild-type APP (SH-SY5Y-APPwt) was established and characterized as previously described 36. HEK293T cells were cultured in DMEM (Gibco, New York, NY, USA) supplemented with 10% fetal bovine serum (FBS; ExCell Bio, Suzhou, China). Parental SH-SY5Y and SH-SY5Y-APPwt cells were grown in MEM/F12 (Procell) supplemented with 15% FBS. All culture media contained 100 U/mL penicillin and 100 µg/mL streptomycin (Gibco). Regular PCR-based testing was conducted to confirm the absence of mycoplasma contamination. All cells were maintained at 37°C under 5% CO2.

### Primary neuronal cell culture

Primary cortical neurons were obtained from postnatal C57BL/6 mice as previously described 37. The growth environment consisted of Neurobasal medium (Gibco) supplemented with 2% B-27 and GlutaMAX (Gibco) on poly-D-lysine-treated plates. To inhibit glial proliferation, cytosine β-D-arabinofuranoside (10 μM) will be added on day 3 *in vitro* (DIV3). A 50% medium replacement schedule was followed every 48 h. Neurons were incubated at 37°C under 5% CO₂ until they were ready for experimental use at DIV7 to DIV8. Medium contained 100 U/mL penicillin and 100 µg/mL streptomycin (Gibco). The primary neuronal were maintained at 37°C under 5% CO2.

### Ubiquitination assay

HEK-293T cells were transiently co-transfected with Myc-DAPK1 and HA-tagged ubiquitin (HA-Ub), which were both constructed in the pcDNA3.1 vector, using transfection reagent (TurboFect) according to the manufacturer's instructions. After 24 h of transfection, cells were treated with CP1 (or vehicle control) for an additional 24 h. Cells were collected, snap-frozen in liquid nitrogen, and stored at -80°C. Cell lysates were prepared in RIPA buffer at 4°C for 30 min and subsequently sonicated. The clarified lysates were incubated with an anti-Myc antibody for 3 h at 4°C for immunoprecipitation. The resulting immunocomplexes were captured using Protein A/G magnetic beads, washed extensively, and eluted for Western blotting analysis to assess DAPK1 ubiquitination using an anti-HA antibody.

### Cell viability assay

To evaluate cell viability, we plated mouse primary cortical neurons in poly-D-lysine-treated 96-well plates (10,000 cells/well). After attachment, the cells were treated with serial concentrations of CP1 for 24 h at 37°C in 5% CO₂. Subsequently, cell viability was assessed using a CCK-8 assay kit according to the manufacturer's instructions. Briefly, 10 μL of CCK-8 reagent was added to each well and incubated for 30 min. A microplate reader (Thermo Fisher Scientific) was used to measure the optical density (OD) at 450 nm, and the data are expressed relative to the control values.

### Hemolysis assay

Hemolysis of CP1 was evaluated using a standardized protocol 38. Briefly, fresh mouse blood was centrifuged (3,000 × g, 10 min, 4°C) to collect erythrocytes, which were subsequently washed and prepared as a 2% (v/v) saline suspension. We mixed this suspension with equal volumes of CP1 at varying concentrations (100-1600 μg/mL) and incubated the mixture for 3 h at 37°C. After centrifugation, the absorbance of the supernatant was read at 576 nm. Saline and deionized water were used as the 0% (negative) and 100% (positive) hemolysis references, respectively. The final hemolysis percentage was derived from the absorbance values, where any value above 5% indicated potential toxicity. The percentage of hemolysis was calculated using the following formula:Hemolysis (%) = [(OD_sample - OD_negative) / (OD_positive - OD_negative)] x 100%.

### Quantitative real-time polymerase chain reaction (qRT-PCR)

Total RNA was extracted and subsequently reverse-transcribed into cDNA using a Transcriptor First Strand Synthesis Kit (Roche) and HiScript^®^ III RT SuperMix (Vazyme) according to the manufacturers' instructions. We performed quantitative PCR (qPCR) using ChamQ Universal SYBR Master Mix on a QuantStudio Real-Time PCR System (Applied Biosystems, Waltham, MA, USA). The amplification protocol consisted of an initial 3-min denaturation at 95°C, followed by 40 cycles of 95°C (20 s), 60°C (30 s), and 72°C (30 s). The 2^-ΔΔCt^ method was used to calculate relative mRNA levels, which were normalized to the β-actin internal control. Details for all the primers are provided in Supplementary Table S1.

### Animals and stereotaxic surgery

Male and female C57BL/6 mice (7 weeks old) were obtained from Beijing HFK Bioscience Co., Ltd. (Beijing, China). The 5xFAD transgenic mice were purchased from Jackson Laboratory (Bar Harbor, ME, USA). All the mice were kept in an SPF-grade facility with a 12-h circadian rhythm and free access to food and water. After 7 days of acclimation, C57BL/6 mice underwent stereotaxic surgery under isoflurane anesthesia (3% induction/1% maintenance), as previously described 39, 40. In brief, we unilaterally delivered rAAV2/9-P301L or rAAV2/9-eGFP (5.9 × 10^12^ viral genomes (vg)/mL, total volume of 640 nL) into the right hippocampal CA3 region (coordinates: anteroposterior: -2.2 mm; mediolateral: +2.7 mm; dorsoventral: -2.3 mm) using a microinfusion pump (Kd Scientific, Holliston, MA, USA) at 125 nL/min, and the needle remained inserted for 5 min post-injection. Following a 7-day recovery period to allow for initial viral expression, the mice in each treatment group (n=7-11 per group) received intraperitoneal injections of CP1 or vehicle twice per week for 6 weeks. This study was approved by the Fujian Medical University Animal Ethics Committee (IACUC FJMU 2018-053).

### Morris water maze (MWM) test

To investigate spatial learning, we used the MWM test as described previously 40. The experimental setup involved a 120-cm diameter circular tank (50 cm deep) containing opaque water maintained at 24°C, which was conceptually partitioned into four quadrants. On day 1, a visible platform trial was carried out with the platform protruding 1 cm above the surface to facilitate visual recognition. Hidden platform acquisition training occurred on days 2-5 (5 trials/day, 60 s/trial maximum). Mice that reached the platform rested for 5 s; mice that failed to reach the platform were guided and allowed 20 s on the platform. A probe trial on day 6 involved platform removal for 60 s of free swim. All behavioral data, including escape latency, target quadrant time, and swim speed, were captured by a camera and processed using SMART v3.0.06 analysis software (Panlab Harvard Apparatus, Barcelona, Spain). All animals were randomly assigned to treatment groups before the start of the study. Behavioral testing (MWM and Y-maze test), image acquisition, and image quantification were performed by investigators blinded to group allocation. No animals were excluded from behavioral analysis based on performance, as all mice completed the testing protocol.

### Y-maze test

To investigate short-term spatial memory, we employed the Y-maze as described earlier 41. The Y-maze apparatus consisted of three identical arms (38 × 8 × 16.5 cm) positioned at 120° angles to one another. To maintain a consistent testing environment, the maze was situated in a quiet, controlled room with standardized ambient lighting. During the training phase, each mouse was placed at the end of one arm; the second arm blocked as the novel arm, and the third arm was left open as the familiar arm. The mice were permitted to freely explore the starting and familiar arms for 5 min. To eliminate potential olfactory cues before the retrieval trial, the maze was briefly decontaminated with 75% ethanol immediately after the 5-min training period. Subsequently, the partition was removed to initiate the 3-min test phase without any further delay, allowing the mice access to all three arms. Using an overhead camera and SMART v3.0.06 software, we recorded the movements of the animals, focusing on their preference for the novel arm, as defined by the entry frequency and residence time. Data quantification was performed using pre-set automated parameters within the tracking software to ensure an objective evaluation.

### Tissue collection and preparation

After behavioral testing, we administered 1.25% (w/v) tribromoethanol (20 mL/kg) to sedate the mice, followed by systemic transcranial perfusion with chilled PBS. We then rapidly harvested the brain and major peripheral organs, including the lungs, heart, liver, and kidneys, for subsequent analysis. One cerebral hemisphere was fixed in 4% paraformaldehyde (PFA) for histology, while the contralateral hippocampus was flash-frozen in liquid nitrogen and stored at -80°C for molecular analyses. Fixed tissues were paraffin-embedded and sectioned at 3-5 μm thickness.

### Extraction of soluble and insoluble Aβ fractions

To separate soluble and insoluble Aβ fractions, brain tissues were homogenized in 5 volumes of ice-cold TBS buffer containing protease and phosphatase inhibitors. The homogenates were centrifuged at 100,000 × g for 60 min at 4°C, and the resulting supernatant was collected as the TBS-soluble fraction. The remaining pellets, containing highly insoluble Aβ aggregates, were thoroughly solubilized in 70% formic acid (FA) and centrifuged again at 100,000 × g for 60 min. The FA-extracted supernatant was neutralized with FA neutralization buffer (1 M Tris, 0.5 M Na₂HPO₄, and 0.05% NaN₃) for subsequent analysis.

### Hematoxylin and eosin (H&E) staining

Paraffin-embedded sections were deparaffinized in xylene, followed by rehydration through a series of decreasing concentrations of ethanol (100% to 75%). The slides were stained with hematoxylin for 5 min, followed by differentiation with 1% acidified ethanol. Eosin counterstaining was performed for 2 min, followed by dehydration, xylene clearing, and mounting in neutral balm. We evaluated the tissue structure and captured representative images using a light microscope (ECHO, San Diego, CA, USA).

### Immunohistochemical (IHC) staining

Deparaffinized and rehydrated sections underwent antigen retrieval. To inhibit endogenous enzyme activity, the sections were treated with 3% hydrogen peroxide, followed by blocking with 10% goat serum. Primary antibody incubation was conducted overnight at 4°C. After a 1-h room-temperature treatment with horseradish peroxidase (HRP)-labeled secondary antibodies (Boster), signals were developed using a DAB substrate kit (MXB Biotechnologies, Fuzhou, China) and counterstained with hematoxylin. Slides were examined microscopically (ECHO), and quantification was performed via ImageJ software. Detailed antibody information is summarized in Table S2.

### Immunofluorescence staining

Deparaffinized and rehydrated sections were subjected to antigen retrieval and blocked with goat serum (Solarbio, Beijing, China) for 1 h, as described previously 21. Primary antibodies were incubated overnight at 4°C in PBS supplemented with 10% goat serum. The sections were incubated with an Alexa Fluor 488/546-labeled secondary antibody (Invitrogen, Carlsbad, CA, USA) for 1 h at room temperature in the dark. After the nuclei were counterstained with Hoechst 33342 (MilliporeSigma), the sections were preserved in antifade mounting medium (Southern Biotech, Birmingham, AL, USA). Confocal or fluorescence imaging was performed on a Zeiss Axio Imager 2 microscope (Oberkochen, Germany), and the fluorescence intensity was quantified using ImageJ software. Detailed antibody information is summarized in Table S2.

### Enzyme-linked immunosorbent assay (ELISA)

The conditioned media, TBS-soluble supernatant, and formic acid-solubilized insoluble pellet were analyzed for Aβ40 and Aβ42 using ELISA kits (Elabscience, Wuhan, China) according to the manufacturer's instructions. Standards/samples (100 μL) were incubated at 37°C for 90 min, followed by biotinylated antibody, HRP-streptavidin, tetramethylbenzidine substrate, and stop solution. Absorbance at 450 nm was measured; concentrations were interpolated from standard curves and normalized to protein content.

### Western blotting

Total protein was extracted from cells or tissues using RIPA lysis buffer supplemented with protease and phosphatase inhibitors. Protein was quantified via a bicinchoninic acid (BCA) assay (Beyotime). Proteins (15-30 μg) were separated by sodium dodecyl sulfate-polyacrylamide gel electrophoresis (SDS-PAGE) and transferred to polyvinylidene difluoride (PVDF) membranes (Merck, Darmstadt, Germany). The membrane was then blocked with 5% BSA or nonfat milk at room temperature for 1 h. Primary antibodies were incubated overnight at 4°C, followed by treatment with HRP-labeled secondary antibodies. The signals were developed with enhanced chemiluminescence (ECL) and imaged on a Bio-Rad ChemiDoc system (Hercules, CA, USA). The band gray values were then quantified with ImageJ software. Detailed antibody information is summarized in Table S2.

### Statistical analysis

Statistical analysis was performed using GraphPad Prism 9.0, with the results from at least three independent replicate experiments expressed as the mean ± standard deviation (SD). Multiple group comparisons were performed via one-way analysis of variance (ANOVA) followed by Tukey's multiple comparison test or Dunnett's post hoc test. For the behavioral escape latency data, two-way repeated-measures ANOVA followed by Tukey's multiple comparisons test. A *p* value < 0.05 was considered to indicate statistical significance.

## Results

### CP1 promotes DAPK1 clearance via ubiquitination and proteasome-dependent proteolysis* in vitro*

**Figure 1:**
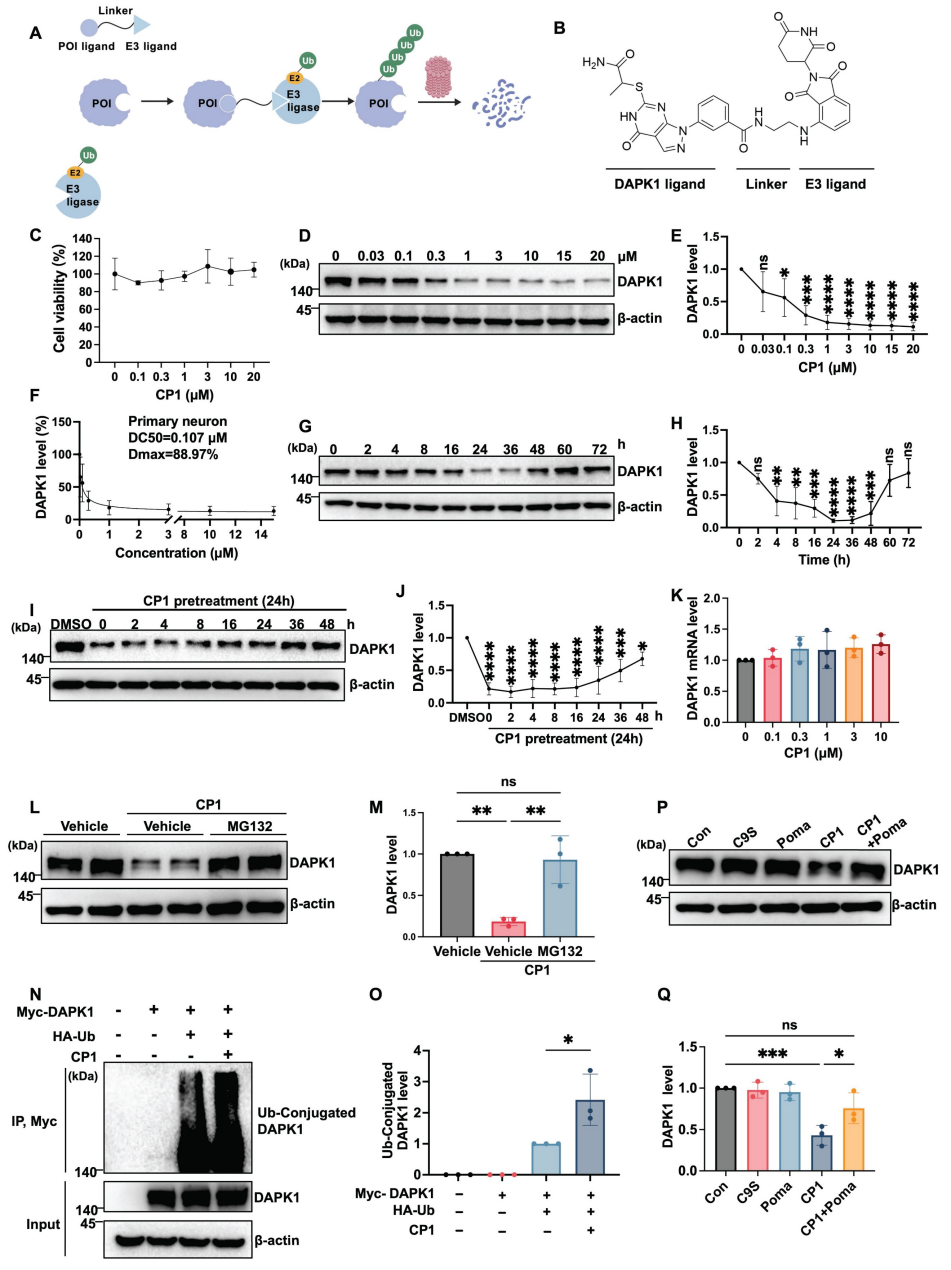
** CP1 induces DAPK1 clearance by promoting its ubiquitination and proteasome-dependent proteolysis *in vitro*.** (A) Schematic diagram illustrating the PROTAC-mediated ubiquitination and proteasomal degradation of DAPK1 through the E1-E2-E3 enzymatic cascade. (B) Chemical structure of CP1, depicting the DAPK1-binding ligand, linker, and recruited E3 ligase ligand. (C) Cell viability assay performed in primary cortical neurons exposed to CP1 (0-20 μM) for 24 h. (D, E) Western blotting analysis of DAPK1 protein levels in primary cortical neurons treated with CP1 (0-20 μM, 24 h); β-actin was used as an internal loading control. *p < 0.05, ***p < 0.001, ****p < 0.0001 and ns, not significant compared with the vehicle control; one-way ANOVA/Dunnett's test. (F) Dose-response analysis of CP1-induced DAPK1 degradation used to determine the DC₅₀ and Dmax value. (G, H) Time-course Western blotting assessment of DAPK1 in neurons treated with 1 μM CP1 for 0-72 h. **p < 0.01, ***p < 0.001, ****p < 0.0001 and ns, not significant compared with the vehicle control; one-way ANOVA/Dunnett's test. (I, J) Western blotting and quantification of DAPK1 recovery following CP1 washout. Neurons were pretreated with CP1 for 24 h, followed by media replacement and incubation for the indicated times (0-48 h). *p < 0.05, ***p < 0.001, ****p < 0.0001 and ns, not significant compared with the vehicle control; one-way ANOVA/Dunnett's test. (K) Quantitative RT-PCR measurement of DAPK1 mRNA in mouse primary cortical neurons treated with CP1 (0-10 μM). Thus, the mRNA expression data are derived from mouse samples only. β-actin was used as an endogenous control. (L, M) Primary cortical neurons were exposed to CP1 for 12 h and subsequently treated with MG132 (10 μM) for an additional 12 h. Western blotting of cell lysates was performed using an anti-DAPK1 antibody, and β-actin was used as an internal control. **p < 0.01 and ns, not significant compared with the vehicle control; one-way ANOVA/Dunnett's test. (N, O) HEK-293T cells were transfected with Myc-DAPK1 and treated with or without HA-ubiquitin and CP1 for 48 h. The cell lysates were immunoprecipitated with an anti-Myc antibody, and HA-tagged ubiquitin conjugated to DAPK1 was detected by Western blotting with an anti-HA antibody. *p < 0.05, compared with the vehicle control; one-way ANOVA/Dunnett's test. Representative blots from three independent experiments are shown. The quantitative data are presented as the mean ± SD (n = 3). (P, Q) Competition assay evaluating DAPK1 levels in primary cortical neurons treated with vehicle control, DAPK1 ligand (C9S), E3 ligand (Poma), CP1, or a combination of CP1 and Poma. For the combination group, neurons were pretreated with Poma (10 μM) for 4 h prior to the addition of CP1 for an additional 24 h. *p < 0.05, ***p < 0.001 and ns, not significant compared with the vehicle control; one-way ANOVA/Dunnett's test.

DAPK1 plays a central role in driving multiple degenerative processes in AD, including synaptic dysfunction, tau hyperphosphorylation, amyloidogenic processing of APP, and neuronal apoptosis 10. To achieve sustained suppression by selectively removing DAPK1 rather than transiently inhibiting its activity, we designed bifunctional PROTAC molecules, which recruit an E3 ubiquitin ligase to DAPK1, thereby promoting its ubiquitination and proteasomal degradation. We designed and synthesized two series of PROTACs, CL and CP, by conjugating the DAPK1-binding ligand HS38 to the CRBN ligands lenalidomide or pomalidomide through linkers of varying lengths. Among all the compounds, CP1 was identified as the most effective degrader of DAPK1 in initial screening and was therefore selected for further evaluation 32. CP1 was synthesized by conjugating a DAPK1-binding ligand (derived from HS38) to an E3 ligase ligand (pomalidomide) through a linker of appropriate length to facilitate ternary complex formation (Figure 1A and B). Detailed synthetic procedures and characterization data are provided in our prior study 32.

We first evaluated the cytotoxicity of CP1 in primary cortical neurons. CCK-8 assays revealed that CP1 at concentrations up to 20 μM did not reduce cell viability (Figure 1C), indicating good neuronal tolerance and suitability for mechanistic studies. To evaluate the degradation efficiency of CP1, we treated primary cortical neurons with increasing concentrations of CP1 for 24 h and measured DAPK1 protein levels by Western blotting. The results revealed that CP1 effectively reduced DAPK1 levels in a dose-dependent manner, exhibiting a half-maximal degradation concentration (DC₅₀) of approximately 0.107 μM and a maximum degradation (Dmax) of 88.97% (Figure 1D-F). To assess the time course of degradation, we treated neurons with 1 μM CP1 for 0 to 72 h. DAPK1 levels progressively decreased between 8 and 72 h of treatment, indicating the sustained degradation durability of CP1 (Figure 1G and H). Furthermore, in order to determine how long DAPK1 suppression lasts after compound removal, we performed washout experiments. Neurons were pretreated with CP1 for 24 h, followed by compound removal, and DAPK1 levels were monitored over time. DAPK1 suppression was robustly sustained for up to 36 h after washout before gradually returning to baseline (Figure 1I and J). To examine whether CP1 affects DAPK1 transcription, we measured DAPK1 mRNA levels by qRT-PCR in mouse primary cortical neurons after CP1 treatment. Thus, the mRNA expression data are derived from mouse samples only. No changes in DAPK1 mRNA were observed across the tested concentrations (Figure 1K), confirming that its activity is posttranslational and mediated by TPD rather than transcriptional suppression.

To determine whether CP1 acts through the ubiquitin-proteasome system, neurons were cotreated with CP1 and the proteasome inhibitor MG132. MG132 treatment fully reversed the reduction in DAPK1 protein expression (Figure 1L and M), indicating that CP1-mediated clearance depends on proteasomal degradation. Furthermore, immunoprecipitation assays demonstrated that CP1 treatment markedly increased the polyubiquitination of DAPK1 (Figure 1N and O), thereby promoting its degradation in a ubiquitin-dependent manner. To further determine whether CP1-induced DAPK1 degradation depends on E3 ligase engagement, we performed a competition assay. As shown in Figure 1P and Q, treatment with CP1 alone significantly reduced DAPK1 levels compared to vehicle control. In contrast, treatment with the DAPK1 ligand (C9S) or the E3 ligase ligand pomalidomide (Poma) alone did not alter DAPK1 levels, confirming that neither ligand alone affects DAPK1 stability. Expectedly, co-treatment with CP1 and pomalidomide effectively blocked CP1-mediated DAPK1 degradation, indicating that degradation requires engagement of the E3 ligase. These results provide direct evidence that CP1 acts through ternary complex formation and CRBN-dependent recruitment. Collectively, these findings establish CP1 as a potent and selective DAPK1 degrader that functions through a classical PROTAC-like mechanism, providing a molecular tool to dissect DAPK1 biology and its contribution to AD pathogenesis.

### CP1 suppresses APP/tau phosphorylation and Aβ production *in vitro*

**Figure 2:**
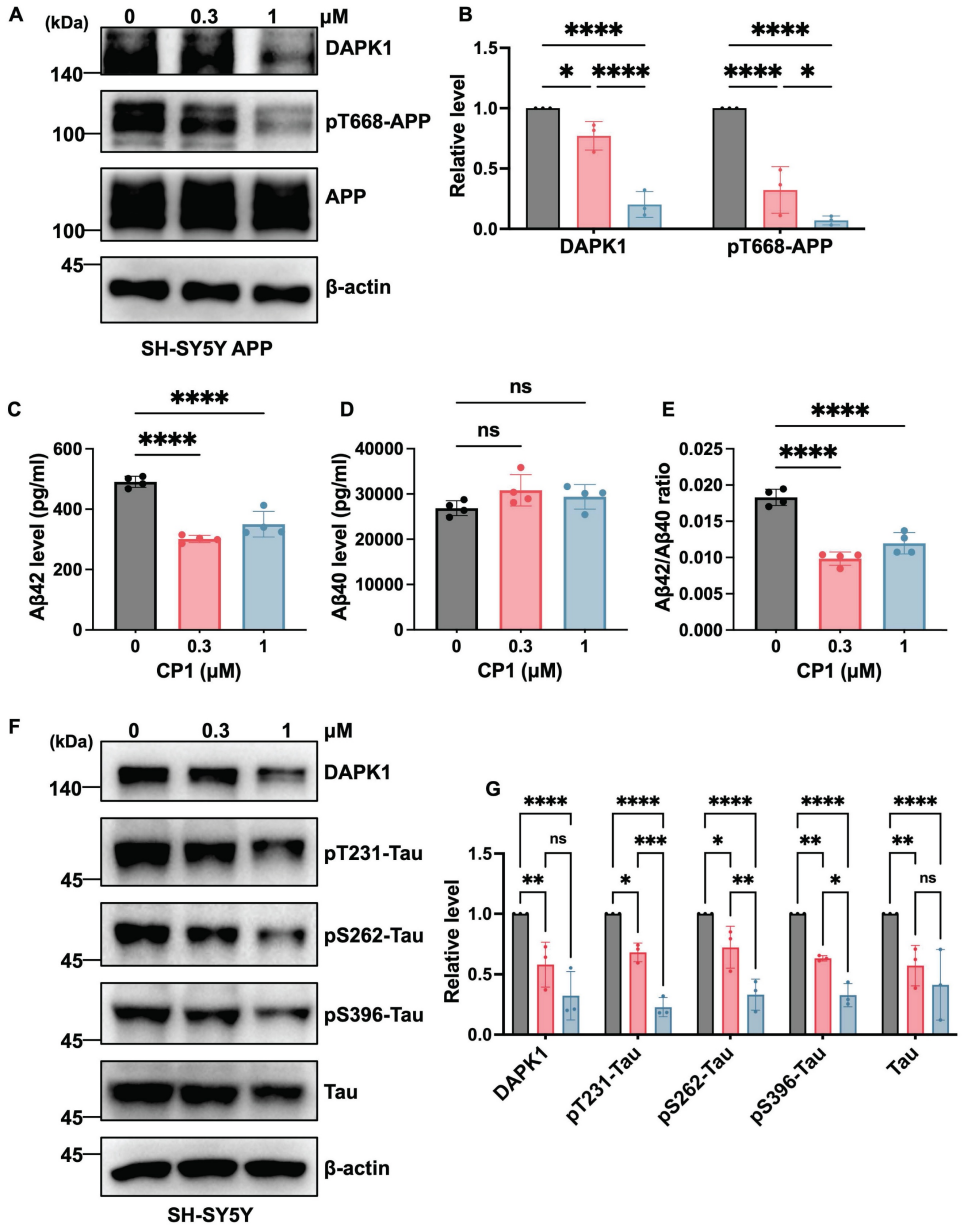
** CP1 reduces tau/APP phosphorylation and Aβ production *in vitro*.** (A, B) Western blotting analysis of DAPK1, phosphorylated APP at Thr668 (pT668), and total APP in SH-SY5Y-APPwt cells treated with CP1 (0-1 μM) for 24 h. β-actin was used as a loading control. *p < 0.05, ****p< 0.0001 for pairwise comparisons among all groups; one-way ANOVA followed by Tukey's multiple comparisons test. (C-E) ELISA quantification of Aβ42 levels, Aβ40 levels, and the Aβ42/Aβ40 ratio in conditioned media collected from SH-SY5Y-APPwt cells exposed to CP1 for 24 h. ****p< 0.0001, ns, not significant, for pairwise comparisons among all groups; one-way ANOVA followed by Tukey's multiple comparisons test. (F, G) Western blotting analysis of DAPK1, phosphorylated tau species (pThr231, pSer262, and pSer396), and total tau in SH-SY5Y cells treated with CP1 (0-1 μM, 24 h). *p < 0.05, **p < 0.01, ***p < 0.001, ****p< 0.0001 and ns, not significant for pairwise comparisons among all groups; one-way ANOVA followed by Tukey's multiple comparisons test. Representative images from three independent experiments are shown. The quantitative data are presented as the mean ± SD (n = 3).

DAPK1 contributes to AD pathogenesis by concurrently modulating tau and APP phosphorylation. Our previous work revealed that DAPK1 interacts with APP through its death domain and upregulates JNK3-dependent APP phosphorylation at Thr668 36. This phosphorylation facilitates the endosomal translocation of APP and increases its colocalization with the β-secretase beta-site amyloid precursor protein cleaving enzyme 1 (BACE1), ultimately shifting APP processing toward the amyloidogenic pathway and ultimately promoting the amyloidogenic cleavage of APP 42. Moreover, DAPK1 aggravates tau phosphorylation at multiple disease-associated sites, either through direct phosphorylation or by modulating other kinases 17, 43. This increased phosphorylation contributes to microtubule destabilization and facilitates the formation of tau aggregates 10. To investigate whether the CP1-mediated degradation of DAPK1 modulates APP related pathologies, we first assessed APP phosphorylation in SH-SY5Y cells overexpressing human APP. Following 24 h of CP1 treatment (0, 0.3 and 1 μM), Western blotting analysis revealed a marked reduction in DAPK1 protein levels accompanied by decreased APP phosphorylation at Thr668, without changes in total APP abundance (Figure 2A and B). Consistent with these observations, ELISA of conditioned media revealed significant reductions in the levels of secreted Aβ42 and Aβ40 and a decreased Aβ42/Aβ40 ratio (Figure 2C-E). Taken together, these results indicate that CP1-mediated DAPK1 degradation attenuates phosphorylation of APP at Thr668 and Aβ generation, which is consistent with reduced amyloidogenic processing. We next investigated whether CP1 influences the phosphorylation of tau at three AD-related sites, including Thr231, Ser262, and Ser396. These sites have been previously shown to be regulated by DAPK1 43-45 and are known to contribute to tau aggregation and microtubule destabilization in AD 46, 47. SH-SY5Y cells were exposed to CP1 (0, 0.3 and 1 μM), and tau phosphorylation at these sites was evaluated by Western blotting. CP1 treatment markedly reduced phosphorylation at all three sites (Figure 2F and G). Collectively, these findings indicate that CP1 suppresses DAPK1-driven APP amyloidogenesis and tau hyperphosphorylation, thereby simultaneously modulating two major molecular cascades that are involved in AD pathogenesis.

### CP1 mediated AD pathology mitigation is DAPK1 dependent

**Figure 3:**
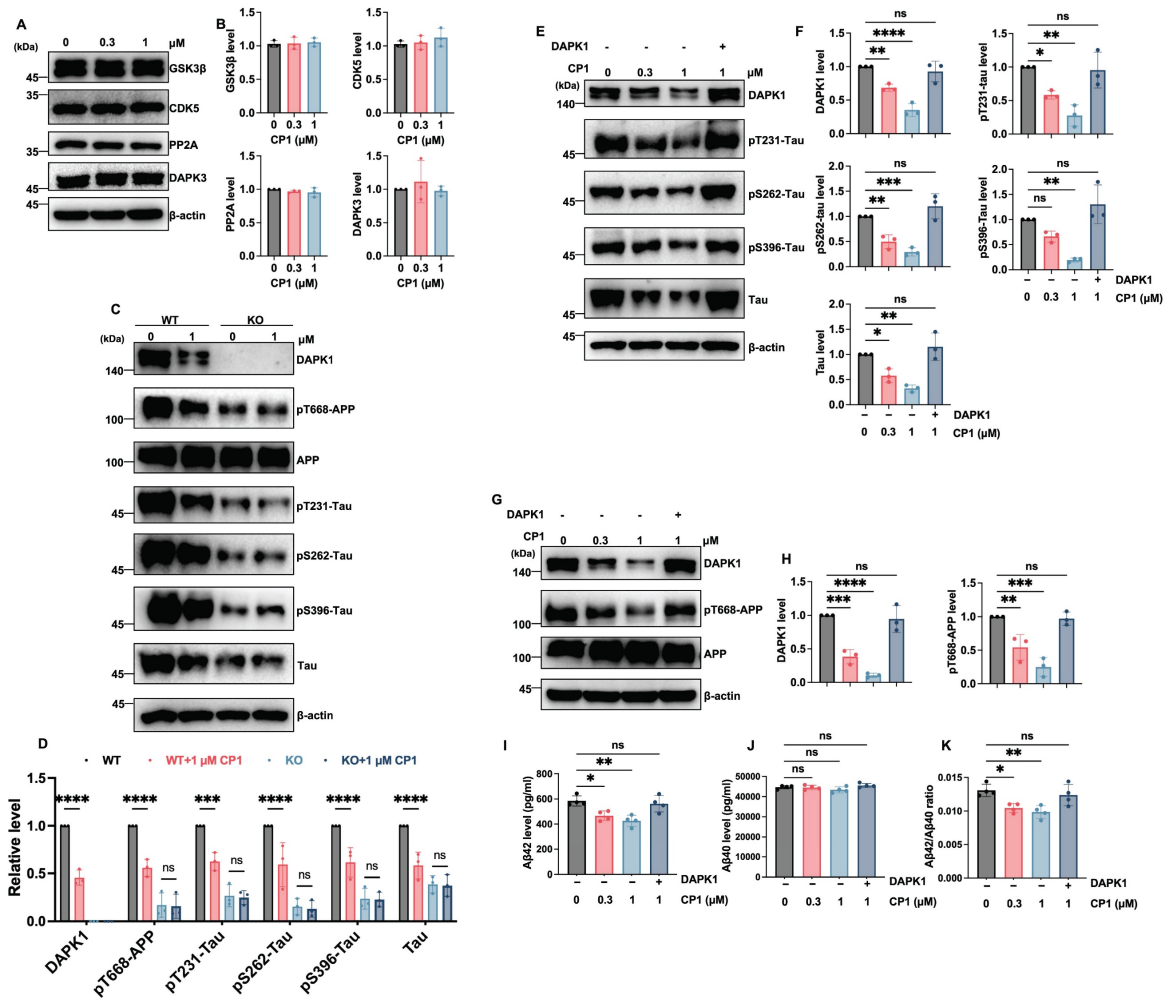
** CP1 mediated AD pathology mitigation is DAPK1 dependent.** (A, B) Western blotting and quantitative analysis of the protein levels of GSK3β, CDK5, PP2A, and DAPK3 in primary cortical neurons treated with CP1 (0, 0.3, and 1 μM). (C, D) WT and DAPK1-knockout (KO) primary cortical neurons were treated with 0 or 1 μM CP1. Representative Western blotting and quantitative analysis of DAPK1, pT668-APP, total APP, pT231-Tau, pS262-Tau, pS396-Tau, and total Tau. ***p < 0.001, ****p < 0.0001, and ns, not significant for pairwise comparisons among all groups; one-way ANOVA followed by Tukey's multiple comparisons test. (E, F) Rescue of tau phosphorylation by exogenous DAPK1. Cells were treated with CP1 (0, 0.3, and 1 μM), and SY5Y cells treated with 1 μM CP1 was overexpressed with DAPK1. Western blotting and quantification of DAPK1, pT231-Tau, pS262-Tau, pS396-Tau, and total tau. *p < 0.05, ** *p* < 0.01, ****p < 0.001, ****p < 0.0001, and ns, not significant for pairwise comparisons among all groups; one-way ANOVA followed by Tukey's multiple comparisons test. (G, H) Western blotting analysis and quantification of DAPK1, pT668-APP, and total APP levels in the DAPK1 rescue experiment. **p < 0.01, ***p < 0.001, ****p < 0.0001, and ns, not significant for pairwise comparisons among all groups; one-way ANOVA followed by Tukey's multiple comparisons test. (I-K) ELISA measurements of secreted Aβ42 (I), Aβ40 (J), and the Aβ42/Aβ40 ratio (K) in the conditioned media from the indicated treatment groups. *p < 0.05, **p < 0.01, and ns, not significant. Representative blots from three independent experiments are shown. The quantitative data are presented as the mean ± SD (n = 3-4).

To further confirm that the inhibitory effects of CP1 on tau pathology are specifically mediated through DAPK1 degradation, we first examined the impact of CP1 on other related kinases. Western blotting analysis showed that CP1 treatment (0.3 and 1 μM) did not alter the protein levels of GSK3β, CDK5, PP2A, or DAPK3 (Figure 3A and B), demonstrating the high target selectivity of CP1. We then utilized DAPK1-knockout (KO) primary cortical neurons, to verify the dependency of these effects on DAPK1. While CP1 treatment dose-dependently reduced the phosphorylation of APP at Thr668 and Tau at Thr231, Ser262, and Ser396 in wild-type (WT) primary cortical neurons, these phosphorylated proteins remained largely unchanged in DAPK1-KO cells following CP1 exposure (Figure 3C and D). These data suggest that the attenuation of APP and tau phosphorylation by CP1 is DAPK1 dependent. To provide definitive evidence, we performed a rescue experiment by overexpressing DAPK1 in CP1-treated cells. We observed that the CP1-induced reduction in tau phosphorylation at Thr231, Ser262, and Ser396 was significantly reversed upon DAPK1 replenishment (Figure 3E and F). Similarly, in SH-SY5Y-APPwt cells the downregulation of DAPK1 and pT668-APP by CP1 was effectively restored by exogenous DAPK1 expression (Figure 3G and H). Furthermore, ELISA results showed that the CP1-mediated decrease in secreted Aβ42 and Aβ40 levels, as well as the Aβ42/Aβ40 ratio, was significantly neutralized by DAPK1 overexpression (Figure 3I-K).

### CP1 reduces amyloid plaques in 5xFAD mice

One of the major challenges in developing therapeutic agents for AD is achieving efficient penetration across the blood-brain barrier (BBB). To assess whether CP1 could reach the central nervous system, we analyzed brain lysates by liquid chromatography-mass spectrometry (LC-MS) and readily detected intact CP1, confirming BBB permeability (Figure S1). CP1 standard showed a distinct peak at retention time of 2.21 min with the corresponding m/z 658.16 and relative abundance of 100%. Similarly, brain extract from a CP1-treated mouse showed a peak at the same retention time with matching m/z, confirming the presence of CP1 in the central nervous system. To evaluate the *in vivo* efficacy of CP1, we used female 5xFAD transgenic mice, a well-established AD model that exhibits early-onset Aβ deposition, neuronal loss, and progressive cognitive decline 48. Given the higher disease incidence and amyloid burden observed in women, female mice were selected to better reflect sex-dependent susceptibility to AD 33. Before systemic administration, the hemolytic potential of CP1 was examined* in vitro* using mouse erythrocytes. Across a broad concentration range, CP1 induced neither membrane rupture nor hemoglobin release (Figure S2A), indicating the absence of acute red blood cell toxicity. Female 5xFAD mice (six months old) were then administered CP1 intraperitoneally (10 or 20 mg/kg) twice weekly for five weeks.

**Figure 4:**
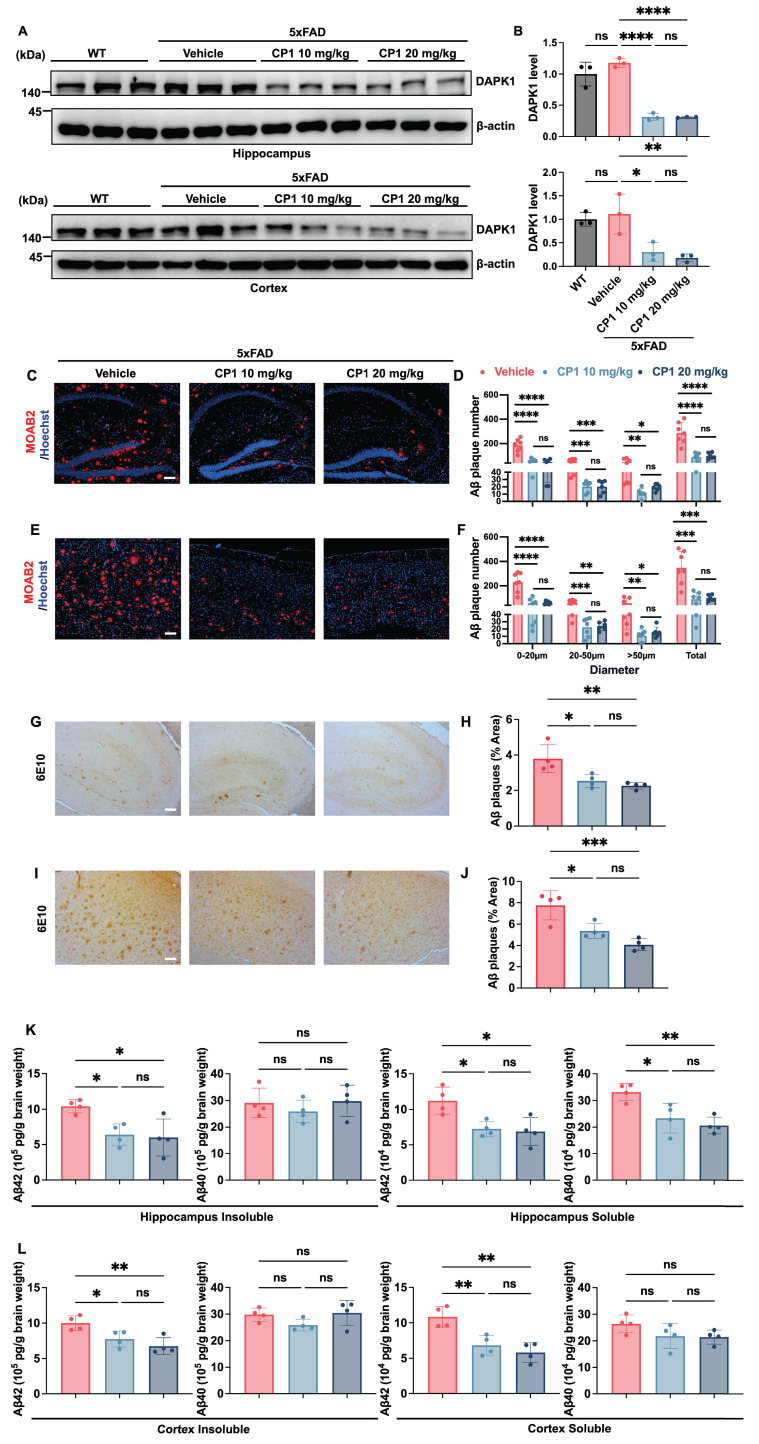
** CP1 reduces the size and number of Aβ plaques in 5xFAD mice.** (A, B) Six-month-old female WT and 5xFAD mice were treated intraperitoneally with either vehicle or CP1 (10 or 20 mg/kg) for five weeks. After the treatment period, hippocampal and cortical lysates were prepared and subjected to Western blotting analysis using an anti-DAPK1 antibody. β-actin was used as a loading control. *p < 0.05, **p < 0.01, ****p< 0.0001, ns, not significant, for pairwise comparisons among all groups; one-way ANOVA followed by Tukey's multiple comparisons test. The quantitative data are presented as the mean ± SD (n = 3). (C, D) Representative immunofluorescence images (C) and quantification (D) of Aβ plaques in the hippocampus stained for MOAB2 (red) and counterstained with Hoechst 33342 (blue). Plaque counts were categorized by diameter (0-20 μm, 20-50 μm, and >50 μm) and total plaque number. The values of plaque counts were quantified per brain region. Scale bar, 100 μm (n = 6-7 mice per group). *p < 0.05, **p < 0.01, ***p < 0.001, ****p< 0.0001 and ns, not significant for pairwise comparisons among all groups; one-way ANOVA followed by Tukey's multiple comparisons test. (E, F) Representative immunofluorescence images (E) and quantification (F) of Aβ plaques in the cortex stained for MOAB2 and counterstained with Hoechst 33342. Plaque counts were categorized by diameter (0-20 μm, 20-50 μm, and >50 μm) and total plaque number. The values of plaque counts were quantified per brain region. Scale bar, 100 μm (n = 6-7 mice per group). *p < 0.05, **p < 0.01, ***p < 0.001, ****p< 0.0001 and ns, not significant for pairwise comparisons among all groups; one-way ANOVA followed by Tukey's multiple comparisons test. (G, H) Immunohistochemical staining for Aβ in the hippocampus of vehicle and CP1 treated 5xFAD mice using an anti-6E10 antibody. (I, J) Immunohistochemical staining of Aβ in the cortex using an anti-6E10 antibody. Scale bar, 100 μm (n = 4 mice per group). *p < 0.05, **p < 0.01, ***p < 0.001, and ns, not significant for pairwise comparisons among all groups; one-way ANOVA followed by Tukey's multiple comparisons test. (K, L) ELISA quantification of Aβ40 and Aβ42 levels in the insoluble and soluble fractions of the hippocampus and cortex from vehicle and CP1treated 5xFAD mice. *p < 0.05, **p < 0.01, and ns, not significant for pairwise comparisons among all groups; one-way ANOVA followed by Tukey's multiple comparisons test. The quantitative data are presented as the mean ± SD (n = 4). To ensure anatomical consistency, representative sagittal sections were selected at a consistent lateral level (approximately 1.56-1.80 mm from the midline). For each subject, one representative field containing the region of interest (hippocampus or cortex) was captured. To minimize technical variability, all images were acquired using identical exposure settings. Image processing was performed by an investigator masked to treatment groups using standardized ImageJ workflows. For MOAB-2 staining: The average plaque size was calculated. For 6E10 staining: The area fraction (%) was measured.

Western blotting of brain lysates revealed that CP1 treatment significantly reduced DAPK1 protein levels in both the hippocampus and the cortex (Figure 4A and B), demonstrating effective target engagement in the brain. To evaluate amyloid pathology, brain sections were immunostained with the Aβ-specific antibody MOAB2 (Figure 4C and E). To further characterize plaque morphology, we quantified plaque diameter distribution under standardized image- analysis conditions and categorized the plaques into small (0-20 μm), medium (20-50 μm), and large (>50 μm) groups. CP1 significantly decreased plaque counts in all three size categories in the hippocampus and cortex (Figure 4D and F), indicating that it suppresses both the initiation of new plaques and the growth of existing plaques. This reduction in amyloid deposition was further verified by immunohistochemistry with the Aβ antibody 6E10, which revealed a pronounced reduction in the amyloid plaque burden in the hippocampal and cortical regions (Figure 4G-J). To further quantify the impact of CP1 on different forms of amyloid, brain tissues were homogenized and centrifuged to separate the soluble and insoluble fractions. The concentrations of Aβ40 and Aβ42 in these fractions were subsequently measured by ELISA. In the hippocampus, CP1 treatment led to a significant reduction in both insoluble and soluble Aβ42 levels, as well as soluble Aβ40 levels, while insoluble Aβ40 remained relatively stable (Figure 4K). Similarly, in the cortex, CP1 administration significantly lowered the concentrations of insoluble Aβ42 and soluble Aβ42, although no significant changes were observed in Aβ40 levels (Figure 4L). Notably, the consistent reduction in Aβ42, the more aggregation-prone and neurotoxic isoform-across both regions and fractions underscores the potent therapeutic potential of CP1 in mitigating amyloid-associated pathology. These results support the conclusion that CP1-mediated degradation of DAPK1 attenuates amyloid plaques *in vivo*. Importantly, CP1 administration did not cause systemic or organ toxicity. Serum biochemical markers, including creatinine (CRE), alanine aminotransferase (ALT) and aspartate aminotransferase (AST), remained unchanged. Moreover, H&E staining of major organs, including the heart, liver, lung and kidney, revealed no histopathological abnormalities (Figure S2B-E). Collectively, these results demonstrate that CP1 is brain penetrant, induces target-specific degradation of DAPK1 *in vivo*, and effectively mitigates amyloid plaques without detectable systemic toxicity, supporting its potential as a disease-modifying therapy for AD.

### CP1 alleviates neuropathology in 5xFAD mice

**Figure 5:**
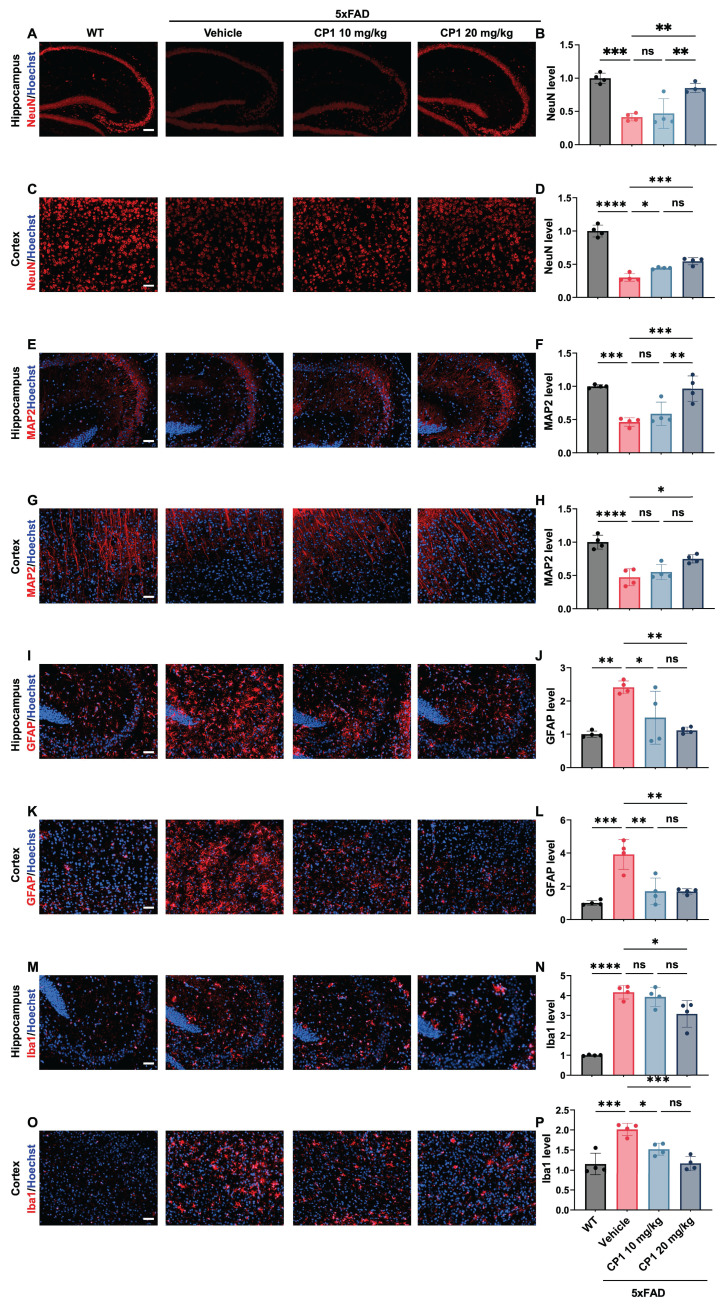
** CP1 alleviates neuropathology in 5xFAD mice.** (A-D) Representative images of immunofluorescence staining of hippocampal and cortex sections from female WT and 5xFAD mice treated with vehicle or CP1 (10 or 20 mg/kg, i.p.) for 5 weeks by anti-NeuN antibodies (red). The cell nuclei were stained with Hoechst 33342 (blue). Scale bar, 50 μm (n = 4 mice per group). *p < 0.05, **p < 0.01, ***p < 0.001, ****p < 0.0001 and ns, not significant for pairwise comparisons among all groups; one-way ANOVA followed by Tukey's multiple comparisons test. (E-H) Representative images of immunofluorescence staining of MAP2 (red) and Hoechst 33342 staining (blue) in the hippocampal and cortical regions. Scale bar, 50 μm (n = 4 mice per group). *p < 0.05, **p < 0.01, ***p < 0.001, ****p < 0.0001 and ns, not significant for pairwise comparisons among all groups; one-way ANOVA followed by Tukey's multiple comparisons test. (I-L) Representative images of immunofluorescence staining of GFAP (red) and Hoechst 33342 staining (blue). Scale bar, 50 μm (n = 4 mice per group). *p < 0.05, **p < 0.01, ***p < 0.001 and ns, not significant, for pairwise comparisons among all groups; one-way ANOVA followed by Tukey's multiple comparisons test. (M-P) Representative images of immunofluorescence staining of Iba1 (red) and Hoechst 33342 staining (blue). Scale bar, 50 μm (n = 4 mice per group). *p < 0.05, ***p < 0.001, ****p < 0.0001 and ns, not significant, for pairwise comparisons among all groups; one-way ANOVA followed by Tukey's multiple comparisons test. (B, D, F, H, J, L, N, P) Quantification of the immunofluorescence intensities of NeuN (B, D), MAP2 (F, H), GFAP (J, L), and Iba1 (N, P) in hippocampal and cortical sections. The quantitative data are presented as the mean ± SD. To ensure anatomical consistency, representative sagittal sections were selected at a consistent lateral level (approximately 1.56-1.80 mm from the midline). For each subject, one representative field containing the region of interest (hippocampus or cortex) was captured. To minimize technical variability, all images were acquired using identical exposure settings. Image processing was performed by an investigator masked to treatment groups using standardized ImageJ workflows.

Excessive activation of DAPK1 contributes not only to amyloid pathology but also to neuronal apoptosis and glial reactivity, two major hallmarks of neurodegeneration in AD 49. To assess whether CP1 exerts neuroprotective effects beyond its anti-amyloid activity, we examined the expression of neuronal and glial markers in 5xFAD mice following five weeks of treatment. Compared with vehicle treatment, CP1 treatment was associated with increased NeuN and MAP2 immunoreactivity in both the hippocampal and cortical regions, as shown by immunofluorescence staining (Figure 5A-H). This observation is consistent with enhanced neuronal integrity and may reflect stabilization of dendritic microtubules and reduced DAPK1-dependent apoptotic signaling. Given that DAPK1 activates p53-and caspase-mediated death pathways in neurons, its degradation by CP1 may alleviate neuronal stress and help sustain synaptic connectivity 50. In addition, the aberrant proliferation of astrocytes and microglia is observed in 5xFAD mice 51, 52. Reactive gliosis is strongly associated with synaptic loss and neurotoxicity 53. The astrocytic marker GFAP and the microglial marker Iba1 are markedly upregulated in response to brain injury or Aβ deposition 54, 55. Immunofluorescence staining revealed significantly fewer GFAP and Iba1 signals in the hippocampus and cortex of CP1-treated mice than in those of vehicle control mice (Figure 5I-P), indicating that compared with vehicle, CP1 effectively attenuated glial activation. Taken together, these results indicate that CP1 preserves neuronal integrity and suppresses excessive glial activation. This coordinated protection of both the neuronal and glial compartments highlights CP1 as a promising therapeutic candidate capable of targeting multiple pathological processes in AD.

### CP1 treatment reduces tau hyperphosphorylation in AAV-hTau-P301L mice

**Figure 6:**
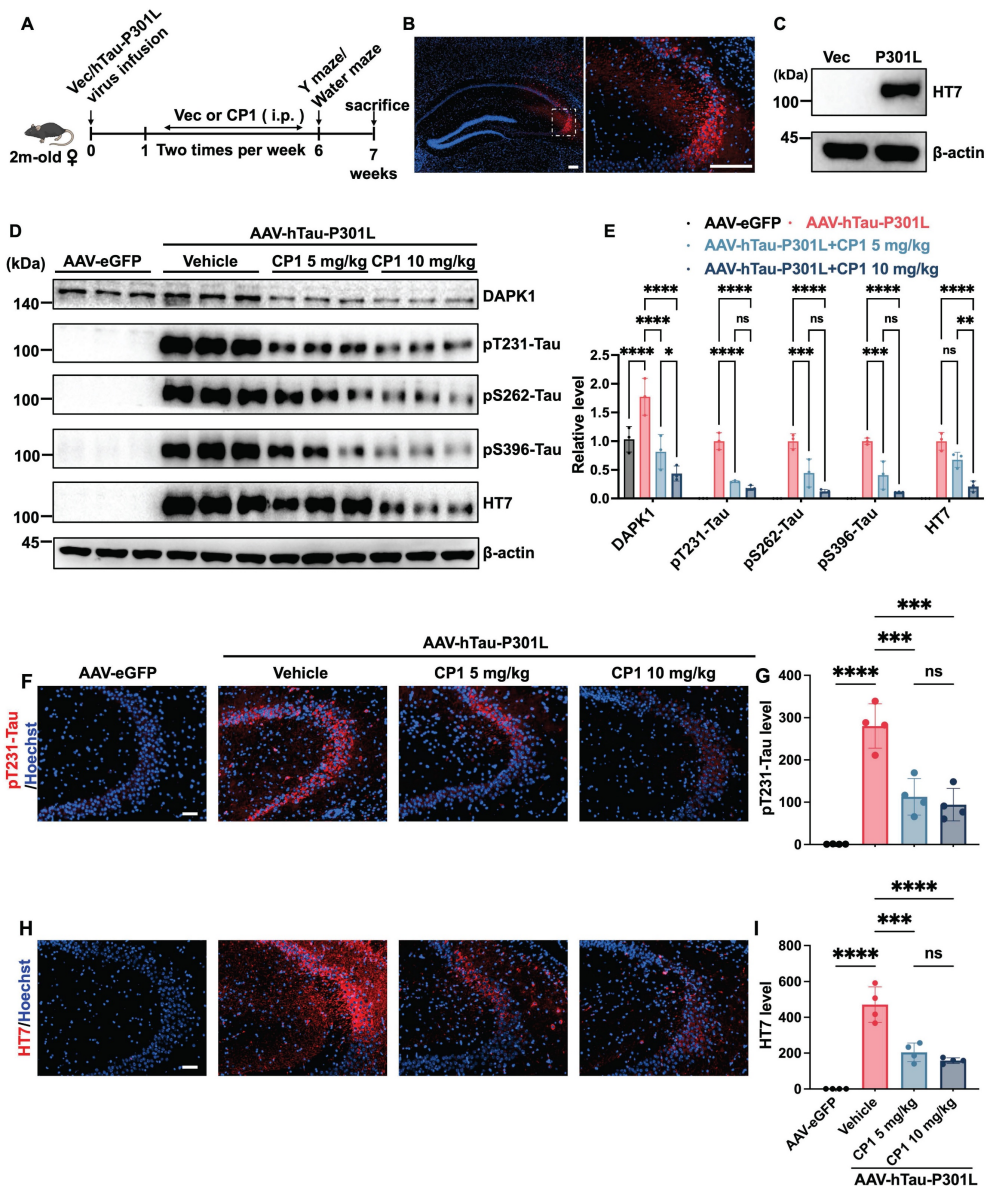
** CP1 treatment reduces tau hyperphosphorylation in AAV-hTau-P301L mice.** (A) Experimental timeline showing the stereotaxic injection of AAV-hTau-P301L or AAV-eGFP into the hippocampus of 2-month-old female C57BL/6 mice, which were randomly divided into four groups (n=11/group) (AAV-eGFP, AAV-hTau-P301L vehicle, AAV-hTau-P301L 5 mg/kg CP1, and AAV-hTau-P301L 10 mg/kg CP1). For histological and immunofluorescence analyses, a subset of mice (n=4/group) was randomly selected for tissue processing. (B) Confirmation of hTau-P301L-eGFP expression in the hippocampal CA3 region by immunofluorescence staining at 7 weeks post-injection with a human tau-specific antibody (HT7). Both the scale bar and the inset scale bar represent 200 μm. (C) Western blotting analysis showing human tau expression (HT7) in hippocampal lysates. (D, E) Representative Western blotting images and quantification of DAPK1, phosphorylated tau (pThr231, pSer262, and pSer396), and total human tau (HT7) in hippocampal lysates. β-actin was used as a loading control. *p < 0.05, **p < 0.01, ***p < 0.001, ****p < 0.0001 and ns, not significant for pairwise comparisons among all groups; one-way ANOVA followed by Tukey's multiple comparisons test. The quantitative data are presented as the mean ± SD (n=3). (F, G) Representative images and quantification of immunofluorescence staining of phosphorylated tau (pThr231, red) and Hoechst 33342 staining (blue) in hippocampal CA3 sections. Scale bar, 50 μm (n = 4 mice per group). ***p < 0.001, ****p < 0.0001 and ns, not significant, for pairwise comparisons among all groups; one-way ANOVA followed by Tukey's multiple comparisons test. (H, I) Representative images and quantification of immunofluorescence staining of total human tau (HT7, red) and Hoechst 33342 staining (blue). Scale bar, 50 μm (n = 4 mice per group). ***p < 0.001, ****p < 0.0001 and ns, not significant, for pairwise comparisons among all groups; one-way ANOVA followed by Tukey's multiple comparisons test. The quantitative data are presented as the mean ± SD. To ensure anatomical consistency with the treatment site, representative coronal sections were selected at the level of the dorsal hippocampus (approximately Bregma -2.2 mm, matching the stereotaxic injection coordinates). For each subject, one representative field containing the target region was captured. To minimize technical variability, all images were acquired using identical exposure settings. Image processing and quantification were performed by an investigator masked to treatment groups using standardized ImageJ workflows.

Tau hyperphosphorylation and accumulation are central features of AD and related tauopathies, leading to microtubule destabilization, axonal transport deficits, and synaptic loss 56. DAPK1 has been identified as an upstream kinase that phosphorylates tau at several disease-relevant epitopes, including Thr231, Ser262, and Ser396, thereby promoting its dissociation from microtubules and facilitating aggregation 44. On the basis of our *in vitro* findings showing CP1-mediated suppression of tau phosphorylation, we next examined whether these effects extend to an *in vivo* tauopathy model. We employed an AAV-mediated model generated by stereotaxic hippocampal CA3 injection of AAV-hTau-P301L-eGFP to study the effect of CP1 on tau pathology *in vivo*. Overexpression of the P301L tau mutant has been shown to induce significant tau hyperphosphorylation in hippocampal neurons across multiple rodent models 57-62. Further studies have confirmed that this overexpression model induces synaptic impairments and cognitive deficits 63. After virus delivery, the mice were treated intraperitoneally with CP1 (5 or 10 mg/kg) twice weekly for five weeks (Figure 6A). These doses were selected based on preliminary studies and are lower than those used in the 5xFAD model (10 or 20 mg/kg), reflecting the younger age of the AAV-hTau-P301L mice (2 months old at start) and the shorter duration of tau pathology relative to the more established amyloid pathology in 5xFAD mice (6 months old). Robust expression of human tau (HT7) in AAV-hTau-P301L-injected mice was confirmed by immunofluorescence and Western blotting (Figure 6B and C). Consistent with the reduction in DAPK1 expression, treatment with CP1 at 5 and 10 mg/kg markedly decreased tau phosphorylation at the Thr231, Ser262, and Ser396 sites (Figure 6D and E). Importantly, total tau levels were reduced only at the 10 mg/kg dose, suggesting a dose-dependent effect. These findings suggest that CP1-mediated DAPK1 degradation limits pathogenic tau phosphorylation and is associated with reduced total tau levels, which may be consistent with enhanced tau clearance. Furthermore, immunofluorescence analysis revealed markedly decreased pThr231-tau immunoreactivity and reduced total human tau staining in the hippocampal CA3 region of CP1-treated mice (Figure 6F-I).

To determine whether these effects are sex independent, we performed identical analyses in male AAV-hTau-P301L mice. CP1 similarly decreased DAPK1 levels and decreased both pThr231-tau levels and total human tau levels, as shown by Western blotting and immunofluorescence (Figure S3A-F). Together, these results demonstrate that the therapeutic benefits of DAPK1 degradation are consistent across sexes. Although the 5xFAD mouse is an established model of Aβ-driven pathology, accumulating evidence indicates that Aβ deposition is sufficient to drive aberrant tau phosphorylation 64. Multiple studies have reported significantly elevated levels of tau phosphorylation at various epitopes in 5xFAD mice, despite the absence of mature neurofibrillary tangles 65, 66. Thus, to determine whether the anti-tau effect of CP1 extends to an Aβ-driven model, we assessed its ability to reduce tau hyperphosphorylation in 5xFAD mice. Consistently, CP1 treatment reduced pThr231-tau levels in the hippocampus and cortex of 5xFAD mice (Figure S4), confirming that DAPK1 degradation effectively suppressed tau hyperphosphorylation at key pathological sites. These findings establish DAPK1 as an upstream driver of tau pathology and highlight the translational promise of its TPD in tau-related neurodegeneration.

### CP1 alleviates neuropathology in AAV-hTau-P301L mice

**Figure 7:**
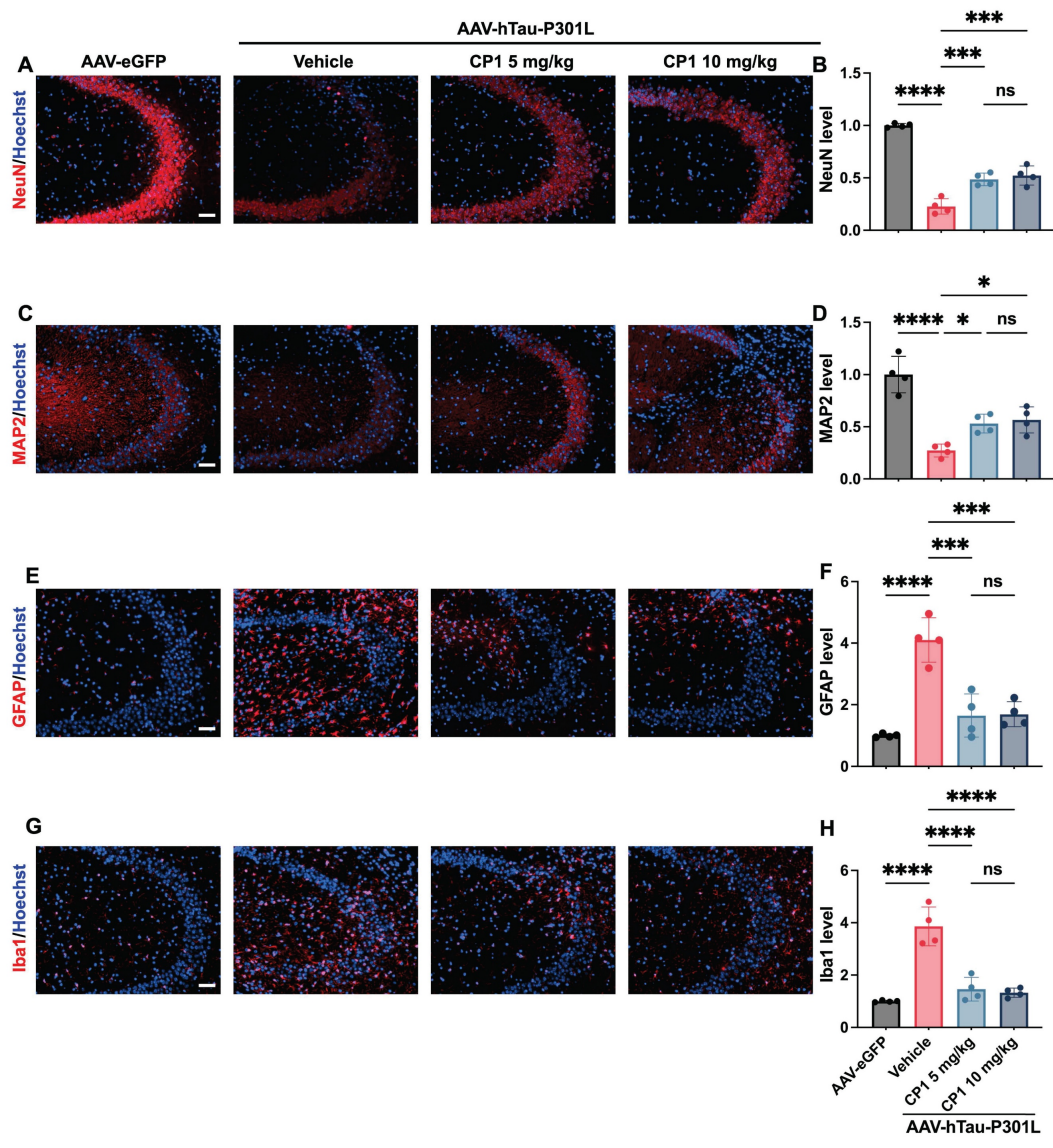
** CP1 alleviates neuropathology in AAV-hTau-P301L mice.** (A, B) Representative images and quantification of immunofluorescence staining of hippocampal sections from female AAV-hTau-P301L mice with anti-NeuN antibodies (red) and Hoechst 33342 (blue). Scale bar, 50 μm (n = 4 mice per group). ***p < 0.001, ****p< 0.0001 and ns, not significant, for pairwise comparisons among all groups; one-way ANOVA followed by Tukey's multiple comparisons test. (C, D) Representative images and quantification of immunofluorescence staining of MAP2 (red) and Hoechst 33342 staining (blue). Scale bar, 50 μm (n = 4 mice per group). *p < 0.05, ****p< 0.0001 and ns, not significant, for pairwise comparisons among all groups; one-way ANOVA followed by Tukey's multiple comparisons test. (E, F) Representative images and quantification of GFAP immunofluorescence staining (red) and Hoechst 33342 staining (blue). Scale bar, 50 μm (n = 4 mice per group). ***p < 0.001, ****p< 0.0001 and ns, not significant, for pairwise comparisons among all groups; one-way ANOVA followed by Tukey's multiple comparisons test. (G, H) Representative images and quantification of Iba1 immunofluorescence staining (red) and Hoechst 33342 staining (blue). Scale bar, 50 μm (n = 4 mice per group). ****p< 0.0001 and ns, not significant, for pairwise comparisons among all groups; one-way ANOVA followed by Tukey's multiple comparisons test. The quantitative data are presented as the mean ± SD. To ensure anatomical consistency with the treatment site, representative coronal sections were selected at the level of the dorsal hippocampus (approximately Bregma -2.2 mm, matching the stereotaxic injection coordinates). For each subject, one representative field containing the target region was captured. To minimize technical variability, all images were acquired using identical exposure settings. Image processing and quantification were performed by an investigator masked to treatment groups using standardized ImageJ workflows.

In P301L-mutant tau-overexpressing mice, hyperphosphorylated tau migrates from axons to dendritic spines, disrupting synaptic integrity and triggering concomitant glial reactivity and neuronal apoptosis 67, 68. DAPK1 promotes cell death via p53- and caspase-dependent cascades and is implicated in the pathogenesis of neurodegenerative diseases 16, 69. Notably, DAPK1 dysregulation drives tau hyperphosphorylation and accumulation and exacerbates neuronal damage by destabilizing microtubule dynamics in AD 10. On the basis of this central role of DAPK1 in tau-mediated toxicity, we evaluated the neuroprotective efficacy of CP1 in AAV-hTau-P301L-injected mice. We analyzed the expression of neuronal and glial markers in female AAV-hTau-P301L mice after five weeks of treatment. Immunofluorescence revealed that compared with the control, CP1 significantly preserved NeuN-positive neuronal density and enhanced MAP2-positive dendritic complexity in the CA3 region of the hippocampus (Figure 7A-D). These morphological improvements suggest that CP1 maintains neuronal integrity and dendritic architecture, which are often compromised by tau hyperphosphorylation and excessive DAPK1 activity. Concurrently, CP1 treatment significantly reduced the expression of GFAP and Iba1, which are markers of astrocytic and microglial activation, respectively (Figure 7E-H). These results indicate that DAPK1 degradation attenuates glial reactivity.

Importantly, the neuroprotective efficacy of CP1 was also validated in male AAV-hTau-P301L mice. These animals exhibited comparable increases in NeuN and MAP2 immunofluorescence, indicating preserved neuronal populations and dendritic integrity (Figure S5A-D). Concurrently, a significant reduction in GFAP and Iba1 immunoreactivity was observed, reflecting attenuated astrogliosis and microglial activation (Figure S5E-H). These parallel findings confirm that the beneficial effects of CP1 are consistent across sexes, underscoring the robustness and generalizability of DAPK1 degradation as a therapeutic strategy. Together, these findings demonstrate that CP1 preserves neuronal activity and diminishes glial activation in tauopathy models.

### CP1 ameliorates cognitive impairment in AAV-hTau-P301L mice

**Figure 8:**
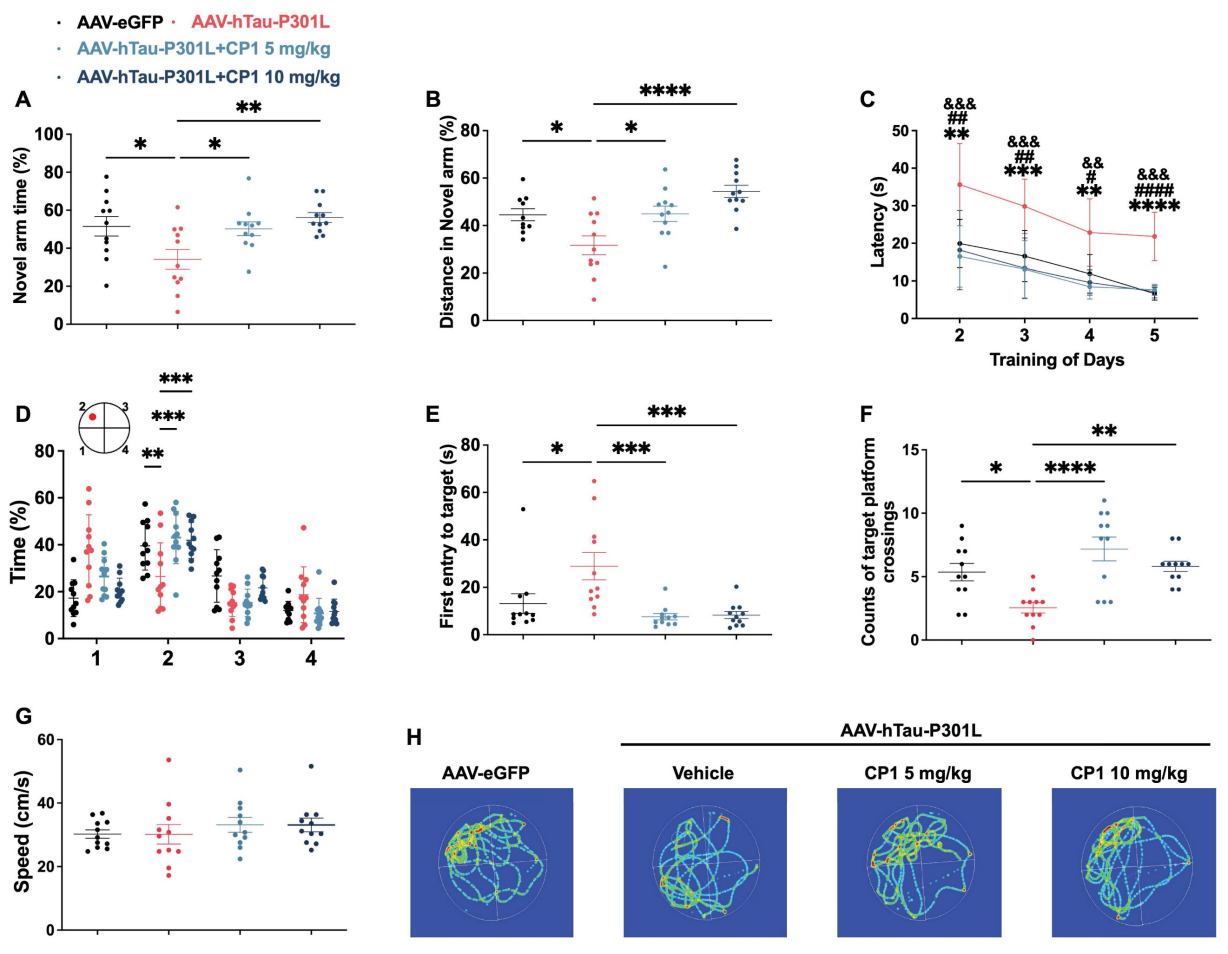
** CP1 ameliorates cognitive impairment in AAV-hTau-P301L mice.** (A, B) Y-maze testing showing the percentage of time spent (A) and distance traveled (B) in the novel arm. (C) MWM training phase assessing spatial learning over consecutive days. (D-F) Probe trial analyses including time spent in the target quadrant (D), latency to first entry (E), and number of platform crossings (F). (G) Average swimming speed during the MWM test. (H) Representative swim trajectories during the probe trial. (A-G) Quantification of behavioral parameters. *p < 0.05, **p < 0.01, ***p < 0.001, and ****p< 0.0001 compared with AAV-hTau-P301L mice treated with the vehicle control. One-way ANOVA followed by Tukey's multiple comparisons test (A, B, D-F); **p < 0.01, ***p < 0.001, and ****p< 0.0001 indicates significant differences between the AAV-hTau-P301L and AAV-hTau-P301L 10 mg/kg CP1 groups; &&*p* < 0.01,&&&*p* < 0.001indicates significant differences between the AAV-hTau-P301L and AAV-hTau-P301L 5 mg/kg CP1 groups; #* p* < 0.05,##* p* < 0.01 and ####* p* < 0.0001 indicate significant differences between the AAV-eGFP and AAV-hTau-P301L vehicle groups, data were analyzed using two-way repeated-measures ANOVA followed by Tukey's multiple comparisons test (C). The quantitative data are presented as the mean ± SD. All experiments were performed using female mice (n = 11 mice per group).

Tau overexpression induces hallmark AD pathologies, including phosphorylated tau accumulation and neurodegeneration, whose progression is tightly associated with decreased brain structure and function, thereby directly linking tauopathy to cognitive deficit 39, 40, 62. Because DAPK1 accumulation and tau hyperphosphorylation are closely linked to neuronal loss and cognitive decline 10, 12, we next assessed whether CP1 treatment could rescue learning and memory in AAV-hTau-P301L mice. In the Y-maze test, compared with control mice, CP1-treated mice showed significantly greater spontaneous alternation and spent more time exploring the novel arm, indicating improved working memory (Figure 8A and B). In the Morris water maze test, CP1 treatment significantly reduced escape latency during training (Figure 8C) and enhanced memory retention in the probe trial, as evidenced by a longer time in the target quadrant, shorter latency to first entry, and increased number of platform crossings (Figure 8D-F). No differences in swimming speed were observed between groups (Figure 8G), ruling out motor effects as a confounding variable. Analyses of representative swimming paths further offered visual confirmation of the improved spatial memory in CP1-treated mice (Figure 8H).

Parallel cognitive improvements were confirmed in male AAV-hTau-P301L mice (Figure S6), suggesting that the therapeutic efficacy of DAPK1 degradation is both robust and sex-independent. Collectively, these findings demonstrate that CP1 rescues learning and memory in tauopathy models through coordinated mechanisms: attenuating tau-driven pathology, preserving synaptic and neuronal integrity, and suppressing neuroinflammatory glial activation. Together, these results support its further development as a disease-modifying therapy for AD.

## Discussion

Traditional kinase inhibitors that function through ATP-competitive binding often face challenges such as poor selectivity, restricted BBB penetration, and an inability to disrupt the non-catalytic scaffolding functions of DAPK1 70. To overcome these limitations, TPD via PROTACs offers a transformative strategy involving the catalytic recruitment of E3 ligases to induce target ubiquitination and subsequent proteasomal degradation, thereby achieving sustained elimination rather than transient inhibition 71, 72. CP1 was rationally designed as a bifunctional PROTAC degrader that links a DAPK1-binding ligand to an E3 ligase recruiter, with the goal of harnessing the ubiquitin-proteasome system to complete DAPK1 removal. This strategy is supported by evidence that DAPK1 increases the phosphorylation of tau at disease-relevant sites, such as Thr231, Ser262 and Ser396, and the phosphorylation of APP at Thr668, promoting tau aggregation, Aβ generation, and neurotoxicity 44, 73. In contrast to conventional inhibitors, PROTAC degraders such as CP1 function catalytically and have the potential to disrupt both enzymatic and scaffolding functions, a dual mechanism that has been exemplified in other successful kinase-targeting degraders 74.

CP1 exhibited no cytotoxicity in primary cortical neurons at concentrations up to 20 μM, suggesting good neuronal tolerance and suitability for mechanistic studies. CP1 induced dose- and time-dependent degradation of DAPK1 in primary cortical neurons, with a half-maximal degradation concentration (DC₅₀) of approximately 0.107 μM. This effect occurred without altering DAPK1 mRNA levels and was completely blocked by the proteasome inhibitor MG132, confirming that degradation is mediated through the ubiquitin-proteasome system. These degradation kinetics are consistent with results from prior studies of DAPK1 showing its role in apoptosis and autophagy, in which genetic knockdown similarly reduces protein levels without affecting transcription 19, 24, 75. To evaluate neuronal tolerance, a key safety requirement for central nervous system therapeutics, we performed cell viability assays. Compared with conventional ATP-competitive inhibitors, which often exhibit off-target toxicity, CP1 exhibited no cytotoxicity at concentrations up to 20 μM 25. CP1-mediated DAPK1 degradation attenuated the phosphorylation of APP and tau, key pathological factors in AD. In both APP-overexpressing cells and primary neurons, CP1 treatment reduced the phosphorylation of APP at Thr668 and decreased the secretion of Aβ42 and Aβ40. These results suggest that the suppression of Aβ generation by CP1 likely results from disruption of the amyloidogenic processing of APP, which is likely achieved by reducing phosphorylation-dependent interactions between APP and the β-secretase complex 42. These findings are consistent with previous findings that DAPK1 inhibition decreased APP amyloidogenic processing and Aβ accumulation 36. However, direct evidence such as sAPPα/sAPPβ ratios, C-terminal fragment or BACE1 activity analysis were not performed. Future studies incorporating these direct measures would help further clarify the underlying mechanism. Moreover, CP1 treatment decreased tau phosphorylation at Thr231, Ser262, and Ser396. These results align with those of our earlier studies demonstrating that DAPK1 inhibition or miRNA-mediated silencing suppresses amyloidogenic processing and normalizes tau phosphorylation 18-24. This evidence highlights the dual-target potential of CP1 in addressing the complex pathology of AD. In this study, we focused primarily on neuronal outcomes. However, DAPK1 is also expressed in glial and vascular cells, and its degradation in these cell types may contribute to the observed reduction in neuroinflammation. Future studies could address whether CP1 acts on these non-neuronal cells by using cell-type-specific conditional knockout models to determine the contribution of DAPK1 in these cells to the overall therapeutic effect.

The efficacy of CP1 was evaluated in female 5xFAD mice, which were chosen for their accelerated Aβ deposition and relevance to sex-specific AD vulnerability, which exhibit greater plaque burden and cognitive decline due to hormonal factors 76. As shown in Figure 3A and B, compared with that in WT controls, DAPK1 protein expression in the brains of 5xFAD mice remained unchanged. These results are consistent with prior literature showing comparable DAPK1 levels between aged Tg2576 mice overexpressing the APP Swedish mutant and WT mice 77. Another study revealed that DAPK1 activity gradually increases in the mouse hippocampus during aging, while its protein level remains constant. Moreover, this age-dependent DAPK1 activation promotes cognitive impairment in aged mice 78. Thus, although DAPK1 expression was not elevated in 5xFAD mice, increased kinase activity may contribute to AD-like pathology and cognitive deficits in this model.

Systemic administration of CP1 reduced DAPK1 levels in the brain. Quantitative analysis of amyloid plaque morphology using the well-established Aβ antibodies MOAB2 and 6E10 revealed that CP1 treatment led to a significant decrease in both plaque number and plaque area, suggesting a shift toward a less aggregated state and an overall attenuated amyloid burden. The histological analysis of amyloid plaques in the 5xFAD model was based on a relatively small cohort (n = 4 mice/group). While the observed effects were robust and statistically significant, future studies with larger or independent cohorts would further strengthen the robustness of these findings. In addition to mitigating amyloid pathology, CP1 produced comprehensive neuroprotective effects in 5xFAD mice. This was evidenced by the preservation of neurons and dendrites coupled with a significant decrease in reactive astrocytes and microglia, which contributed to synaptic loss 79, 80. Quantification of these markers via immunofluorescence provided a composite view of neuroprotection, which is relevant because glial activation is both a biomarker of progression and a consequence of neuronal injury 81. Mechanistically, CP1-induced DAPK1 degradation may protect synapses by stabilizing the cytoskeleton and preventing microtubule destabilization and is further implicated in the inhibition of DAPK1-driven inflammatory and apoptotic cascades. This dual action is supported by earlier findings that DAPK1 ablation mitigates inflammation in neurodegeneration 82.

In the AAV-hTau-P301L tauopathy model, which isolates tau-driven pathology independently of Aβ, CP1 reduced total tau and tau phosphorylation at Thr231, Ser262, and Ser396, as determined through biochemical and histological assays. Its efficacy was consistent in males, confirming that DAPK1-targeted degradation is sex-independent and supporting the generalizability of our results. Moreover, in AAV-hTau-P301L mice of both sexes, CP1 maintained neuronal integrity and attenuated neuroinflammation, which were key pathologies selected for their established correlation with functional decline in tauopathies. Quantitative analysis revealed that the NeuN and MAP2 signal intensities were maintained at approximately 20% and 25%, respectively, in female AAV-hTau-P301L mice, whereas males showed an approximately 50% and 40% retention, respectively, relative to vehicle-treated controls. This finding is consistent with prior reports that female P301L tau-transgenic mice undergo more severe neuronal loss and synaptic injury 35, 83. Notably, female P301L tau-transgenic mice exhibit more severe cognitive and motor deficits, which are closely related to greater synaptic damage 83. Furthermore, the quantification of glial activation markers in the AAV-hTau-P301L model revealed approximately 4-fold increases in GFAP and Iba1 expression in female mice relative to controls, whereas males exhibited increases of approximately 6.5-fold and 7.5-fold in GFAP and Iba1 expression, respectively. These data indicate stronger neuroinflammatory responses in male mice, which is consistent with prior reports that 3xTg-AD mice with P301L tau overexpression exhibit age-related dysregulation of neuro-immune-endocrine crosstalk, particularly in males 84. In addition, CP1 treatment at both 5 and 10 mg/kg rescued cognitive deficits in female AAV-hTau-P301L mice, as assessed by increased exploration in the Y-maze and the ability to acquire spatial memory more rapidly in the MWM test, which involves learning and spatial memory tests. These behavioral improvements are consistent with the molecular and histological findings that CP1-mediated degradation of DAPK1 reduced tau phosphorylation (Figure 6D-I), preserved neuronal populations (Figure 7A-D), and suppressed glial activation (Figure 7E-H). These gains align with reduced tau pathology and neuroprotection, supporting the role of DAPK1 as a cognitive modulator 10. However, in male mice, a significant improvement in cognition was observed only at the 10 mg/kg dose, while the 5 mg/kg dose resulted in a non-significant improvement. The absence of significance at the lower dose may be attributable to sample size. Future studies with larger numbers of mice would help determine whether 5 mg/kg can produce statistically significant cognitive rescue.

Although our results underscore the therapeutic promise of CP1, several limitations warrant consideration. First, we evaluated the effects of CP1 on amyloid and tau pathology independently using the 5xFAD and AAV-hTau-P301L models, which helps clarify that DAPK1 degradation can address both core features of AD. However, these models do not fully capture the coexistence of both pathologies seen in human AD. Future studies using models that combine amyloid and tau would provide a more integrated view of the therapeutic potential of CP1. Second, although LC-MS confirmed BBB penetration by CP1, key pharmacokinetic parameters, including systemic exposure, brain concentration over time, regional brain distribution, and residence time within the brain parenchyma, remain to be elucidated. Techniques such as fluorescent labeling, quantitative autoradiography, or detailed time-course pharmacokinetic analysis could be employed to visualize its regional distribution and supporting dose optimization. Third, the overexpression-based models used in this study, including the SH-SY5Y-APP stable cell line and the AAV-hTau-P301L mouse model, have limitations compared to endogenous disease mechanisms, as overexpression may amplify pathological signals and does not fully mimic the chronic nature of human AD. While these models are useful for initial efficacy testing, future studies employing chronic models that recapitulate the full complexity of pathology, such as hTau transgenic model or human induced pluripotent stem cells-derived neurons, will be necessary to determine whether the neuroprotective benefits of CP1 are sustained throughout progressive neurodegenerative stages. Fourth, DAPK1 is known to function as a tumor suppressor in peripheral tissues, raising potential safety concerns for chronic systemic inhibition in AD. Although no overt toxicity was observed during the five-week treatment period, long-term safety requires further evaluation. Future strategies such as brain-targeted delivery or intermittent dosing could help minimize peripheral exposure while maintaining central efficacy. Extended toxicology studies will also be important to assess these risks as development progresses. Fifth, recent work showed that peripheral cancer can actually reduce amyloid pathology in AD models 85. The dual role of DAPK1 adds an important layer of complexity to therapeutic development. Whether chronic DAPK1 degradation for AD treatment exerts distinct effects on the central nervous system versus peripheral tissues remains an open question. Future studies should help answer whether this approach is safe in people with higher cancer risk, and whether certain patients might benefit more than others. Finally, PROTAC-based approaches also come with practical challenges. These include concerns about compound stability, brain delivery, potential immunogenicity with long-term dosing, and overall suitability for chronic administration. Acknowledging both the opportunities and limitations of this strategy helps provide a more balanced perspective on its potential for AD therapy.

## Conclusion

**Figure 9:**
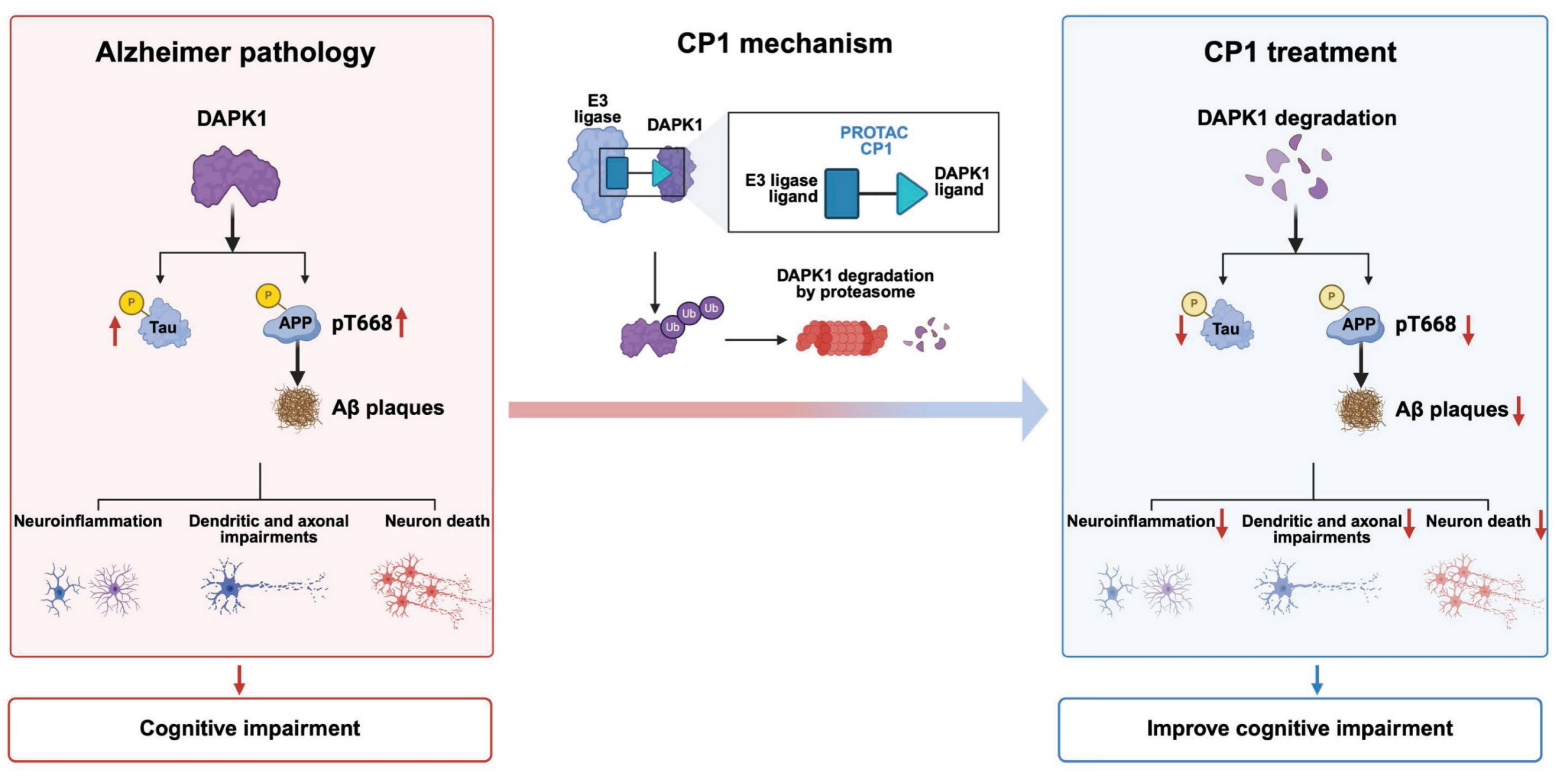
** Schematic model illustrating CP1-mediated DAPK1 degradation and its impact on AD pathology.** In AD pathogenesis, elevated DAPK1 levels correlate with tau hyperphosphorylation, enhanced APP phosphorylation at Thr668, and increased Aβ deposition. These pathological events are accompanied by exacerbated neuroinflammation, dendritic and axonal impairments, and neuronal loss, ultimately leading to cognitive deficits. CP1, a PROTAC targeting DAPK1, recruits an E3 ubiquitin ligase to induce DAPK1 degradation via the ubiquitin-proteasome system. Consequently, depletion of DAPK1 attenuates abnormal tau and APP phosphorylation, reduces Aβ plaque burden, and alleviates subsequent neuropathological damage, which aligns with the observed improvements in cognitive function. The figure was created using BioRender.

In summary, by using DAPK1 as a pivotal upstream driver that promotes amyloidogenic and tau-related pathways in AD, we developed CP1, a brain-permeable PROTAC that selectively recruits DAPK1 for ubiquitin-proteasome-dependent degradation. A proposed working model is presented in Figure 9, illustrating the transition from DAPK1-driven AD pathology to the therapeutic mechanism of CP1. CP1 treatment effectively eliminated DAPK1 protein both *in vitro* and *in vivo*, thereby attenuating APP and tau phosphorylation, reducing Aβ deposition, and preserving neuronal and synaptic integrity in multiple AD mouse models. By broadly mitigating neuroinflammation, protecting dendritic and axonal integrity, and preventing neuronal death, CP1-mediated degradation ultimately ameliorates cognitive impairment (Figure 9). Importantly, these benefits were achieved without overt systemic toxicity or behavioral abnormalities, underscoring the translational potential of DAPK1-targeted degradation. Notably, the dual capacity of CP1 to simultaneously mitigate both amyloid and tau pathology highlights DAPK1 as a unifying therapeutic target whose removal helps restore network homeostasis and cognitive function. This targeted degradation strategy extends beyond conventional kinase inhibition by enabling the elimination of critical upstream hubs that drive convergent neurodegenerative cascades. The dual capacity of CP1 to simultaneously modulate amyloid and tau pathologies highlights DAPK1 as a unifying therapeutic target whose removal restores network homeostasis and cognitive function. TPD offers a distinct advantage over traditional inhibition by eliminating critical upstream hubs that drive multiple neurodegenerative pathways. Future investigations should focus on optimizing the pharmacokinetics, BBB permeability, and long-term safety of DAPK1 degraders and evaluating their efficacy in human-derived neuronal systems and nonhuman primate models. Ultimately, this strategy may pave the way for disease-modifying therapies that address the multifactorial complexity of AD.

## Supplementary Material

Supplementary figures and tables.

## Data Availability

All data generated or analyzed in the present study are available from the corresponding author upon reasonable request.

## Abbreviations

PROTAC: proteolysis-targeting chimera
DAPK1: death-associated protein kinase 1
AD: alzheimer's disease
Aβ: amyloid-β
APP: amyloid precursor protein
MWM: morris water maze
BBB: blood-brain barrier
CRE: creatinine
ALT: alanine aminotransferase
AST: aspartate aminotransferase
NeuN: neuronal nuclei
MAP2: microtubule-associated protein 2
GFAP: glial fibrillary acidic protein
Iba1: ionized calcium-binding adaptor molecule 1
TPD: targeted protein degradation
