# Supplementary material for: A Novel PROTAC Confers a Dual Benefit Against Amyloid and Tau Pathology in Alzheimer's Disease via DAPK1 Degradation
Source: Int J Biol Sci. 2026 Apr 23;22(9):4724–46. doi: 10.7150/ijbs.131465 (PMC13182242; doi:10.7150/ijbs.131465)
Supplement: Supplementary file 1 — Supplementary figures and tables. [file ijbsv22p4724s1.pdf]

Supplementary material

A Novel PROTAC Confers a Dual Benefit Against Amyloid and Tau Pathology in Alzheimer's Disease via DAPK1 Degradation

Table S1. Sequence information of qRT-PCR in the study

Primers-qRT-PCR	5′ - 3′
Mouse DAPK1-F	GCACCCAAATGTCATCACCCT
Mouse DAPK1-R	AAACAGCTCACCTCCTGCAAC
Mouse β-actin-F	GTGACGTTGACATCCGTAAAGA
Mouse β-actin-R	GCCGGACTCA-TCGTACTCC

F, Forward primer; R, Reverse primer

Table S2. Information about antibodies used in the study

Antibody	Dilution	Source	Identifier	RRID	Ref. PMID	LOT
Mouse anti-DAPK1	1:2000 (WB)	Sigma	D2178	AB_259206	32366830	0000113681
Rabbit anti-HA	1:3000 (WB)	Cell Signaling Technology	3724S	AB_1549585	41608571	11
Rabbit anti-pT231-Tau	1:2000 (WB) 1:500 (IF)	Abcam	ab151559	AB_2893278	40762561	1034680-72
Rabbit anti-pS396-Tau	1:2000 (WB)	Abcam	Ab109390	AB_10860822	40762561	1026269-59
Rabbit anti-pS262-Tau	1:2000 (WB)	Invitrogen	44-750G	AB_1502106	38916730	PA5-85654
Mouse anti-Tau (HT7)	1:10000 (WB) 1:500 (IF)	Invitrogen	MN1000	AB_2314654	33712082	ZJ4527065A
Mouse anti-Tau-5	1:10000 (WB) 1:500 (IF)	Invitrogen	AHB0042	AB_1502093	39753133	ZL404703
Rabbit anti-Y188(total APP)	1:5000 (WB)	Abcam	Ab32136	AB_2289606	40838881	1115792-21
Rabbit anti-pAPP(668)	1:3000 (WB)	Cell Signaling Technology	6986S	AB_10831197	39545066	2
Mouse anti-MOAB2	1:200 (IF)	Novus	NBP2-13075	AB_3260692	37358017	D162723-2
Mouse anti-6E10	1:200 (IHC)	Biolegend	803001	AB_2564653	39164276	B428958
Rabbit anti-MAP2	1:200 (IF)	Cell Signaling Technology	4542	AB_10693782	41203647	4
Mouse anti-NeuN	1:500 (IF)	Merck	MAB377B	AB_177621	40318630	4212256
Rabbit anti-GFAP	1:500 (IF)	Abcam	ab7260	AB_305808	40585973	102424-2
Rabbit anti-Iba1	1:500 (IF)	Abcam	ab178847	AB_2832244	40882623	1001575-1
Mouse anti-β-actin	1:40000 (WB)	Sigma	A5441	AB_476744	41443191	0000120485
HRP-conjugated goat anti-rabbit secondary antibody	1:10000	Bio-rad	1706515	AB_11125142	41443191	64601808

Table S2. (Continued)

Antibody	Dilution	Source	Identifier	RRID	Ref. PMID	
HRP-conjugated goat anti-mouse secondary antibody	1:10000	Bio-rad	1706516	AB_11125547	41443191	64643429
Alexa Fluor 546 goat anti-rabbit secondary antibody	1:400	Invitrogen	A11035	AB_2534093	41512733	3219285
Alexa Fluor 546 goat anti-mouse secondary antibody	1:400	Invitrogen	A11030	AB_2534089	41707654	2978791

WB, Western blotting; IF, immunofluorescence; IHC, Immunohistochemistry

A

CP1 standard

Retention time: 2.21 min

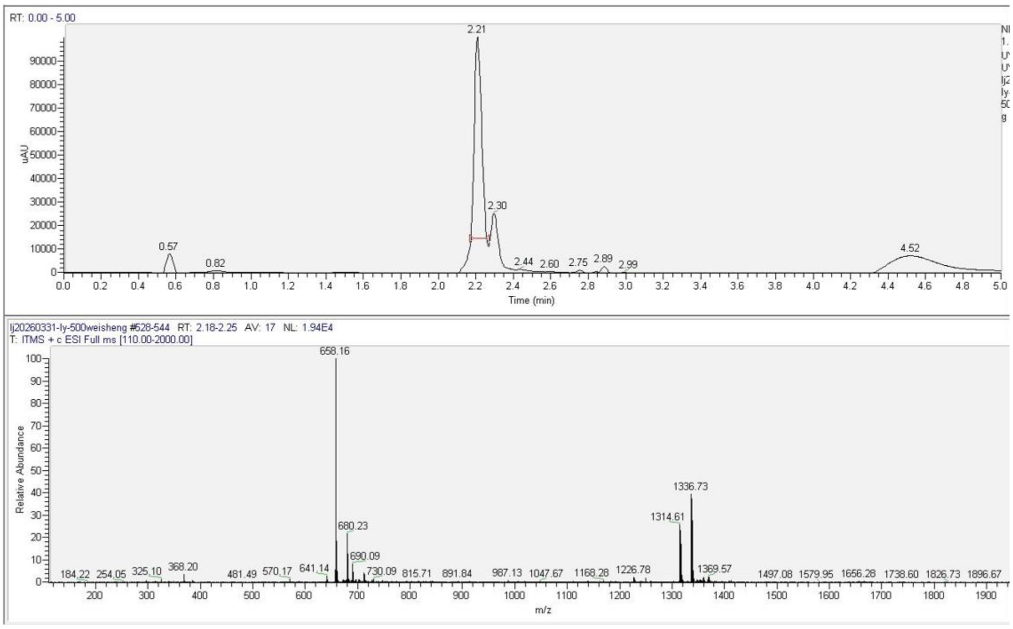

B

CP1-treated brain extract

Retention time: 2.19 min

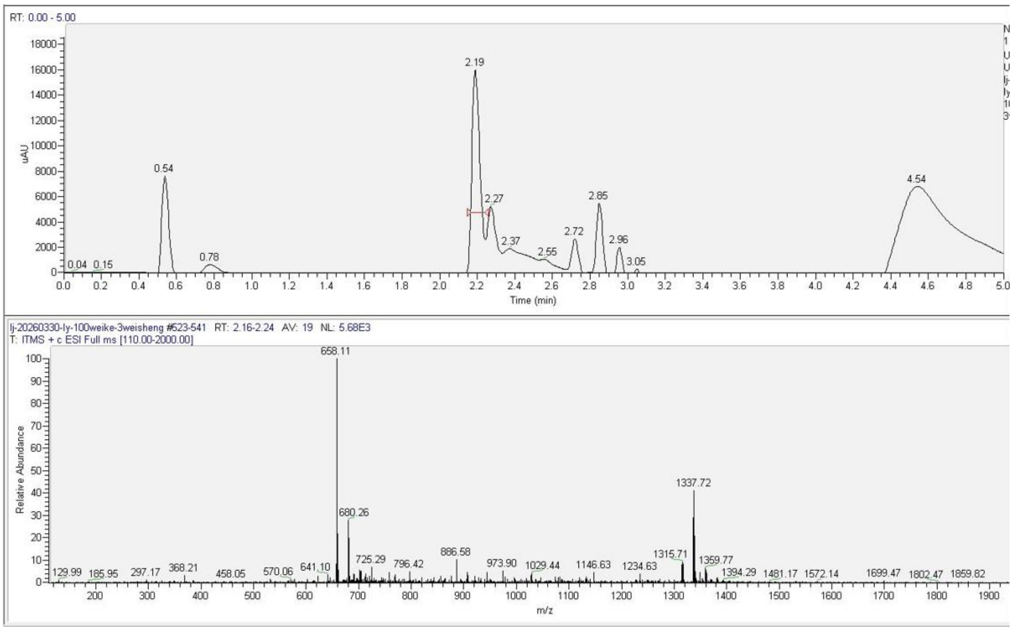

Figure S1. Blood-brain barrier test

(A, B) Representative chromatograms and mass spectra of CP1 in brain extracts from C57BL/6 WT mice. (A) CP1 standard. (B) Brain extract from a CP1-treated mouse.

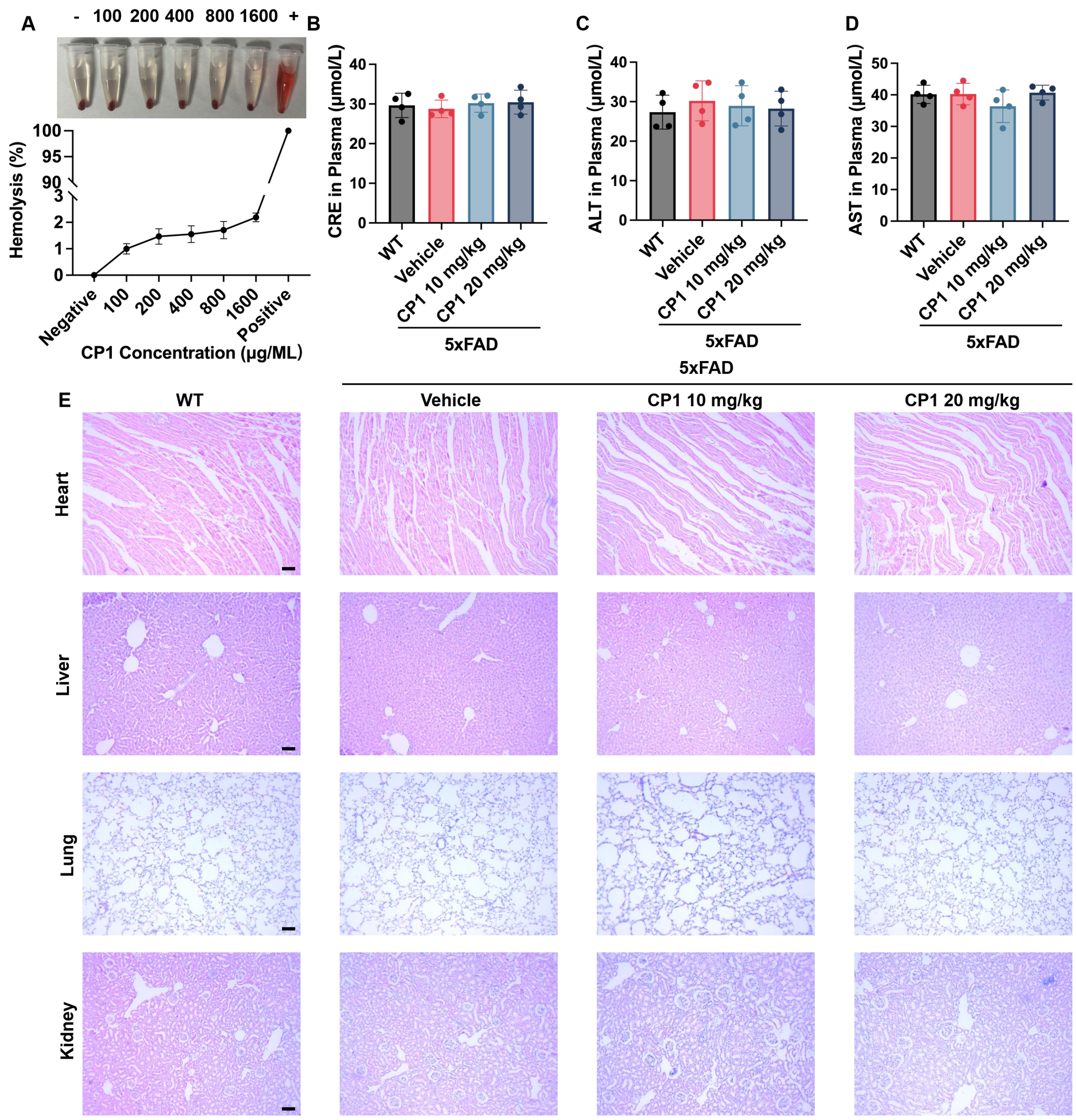

Figure S2. Toxicity assessment of CP1

(A) Experimental setup for the hemolysis assay, in which whole blood collected from C57BL/6J mouse hearts was incubated with increasing concentrations of CP1 (100–1600 μg/mL). Saline and deionized water served as negative and positive controls, respectively. (B-D) Biochemical analysis of plasma obtained from WT and 5xFAD mice treated with vehicle or CP1 (10 or 20 mg/kg), measuring (B) CRE, (C) ALT, and (D) AST levels. (E) Representative H&E staining of major organs, including the heart, liver, lung, and kidney, from treated WT and 5xFAD mice. Scale bar, 50 μm (n = 4 mice per group). Data were analyzed using one-way ANOVA followed by Tukey's multiple comparisons test. Values are presented as mean ± SD.

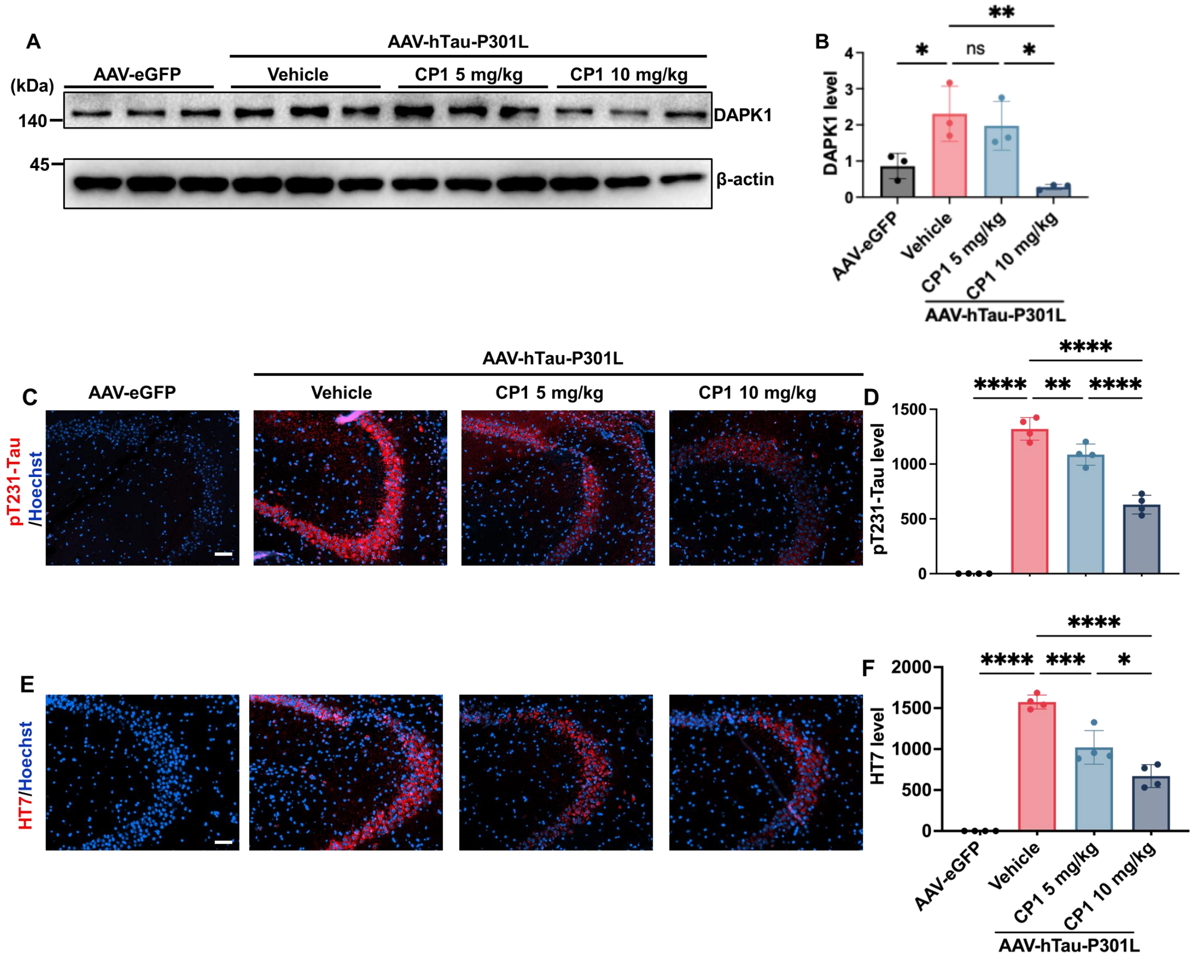

Figure S3. CP1 treatment reduces tau hyperphosphorylation in male AAV-hTau-P301L mice

(A, B) Representative Western blotting images and quantification of DAPK1 in hippocampal lysates. β-actin was used as a loading control. *$p < 0.05$, **$p < 0.01$ and ns, not significant, for pairwise comparisons among all groups; one-way ANOVA followed by Tukey's multiple comparisons test. (C, D) Representative images of immunofluorescence staining of phosphorylated tau (pThr231, red) and Hoechst 33342 staining (blue) in hippocampal CA3 sections. Scale bar, 50 μm (n = 4 mice per group). **$p < 0.01$ and ****$p < 0.0001$ for pairwise comparisons among all groups; one-way ANOVA followed by Tukey's multiple comparisons test. (E, F) Representative images of immunofluorescence staining of total human tau (HT7, red) and Hoechst 33342 staining (blue). Scale bar, 50 μm (n = 4 mice per group). *$p < 0.05$, ***$p < 0.001$ and ****$p < 0.0001$ for pairwise comparisons among all groups; one-way ANOVA followed by Tukey's multiple comparisons test. Representative images from three independent experiments are shown. The quantitative data are presented as the mean ± SD.

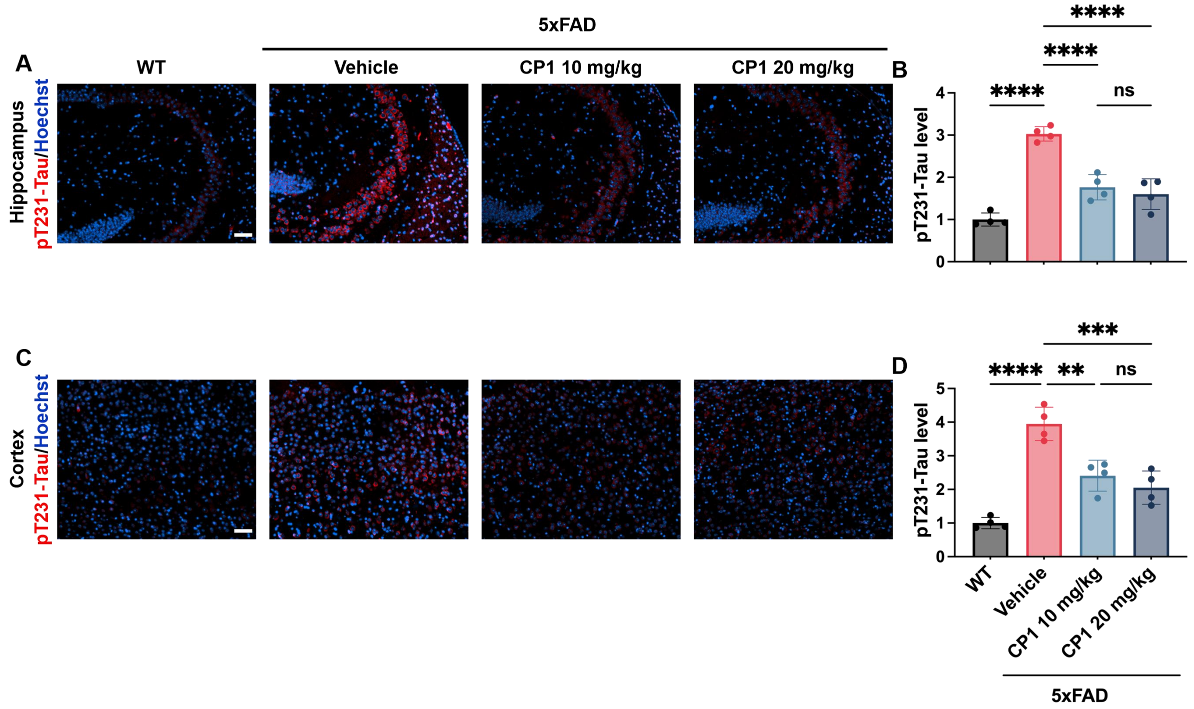

Figure S4. CP1 treatment reduces tau hyperphosphorylation in 5xFAD mouse

(A, B) Representative immunofluorescence images (A) and quantification (B) of phosphorylated tau at Thr231 (pT231-tau, red) in the hippocampal region of female WT and 5xFAD mice treated with vehicle or CP1 (10 or 20 mg/kg). Nuclei were counterstained with Hoechst 33342 (blue). Scale bars, 50 μm (n = 4 mice per group). ****$p< 0.0001$ and ns, not significant, for pairwise comparisons among all groups; one-way ANOVA followed by Tukey's multiple comparisons test. (C, D) Representative immunofluorescence images (C) and quantification (D) of pT231-tau in the cortex of the same treatment groups. Scale bars, 50 μm (n = 4 mice per group). **$p < 0.01$, ***$p < 0.001$, ****$p < 0.0001$ and ns, not significant, for pairwise comparisons among all groups; one-way ANOVA followed by Tukey's multiple comparisons test. The quantitative data are presented as the mean ± SD.

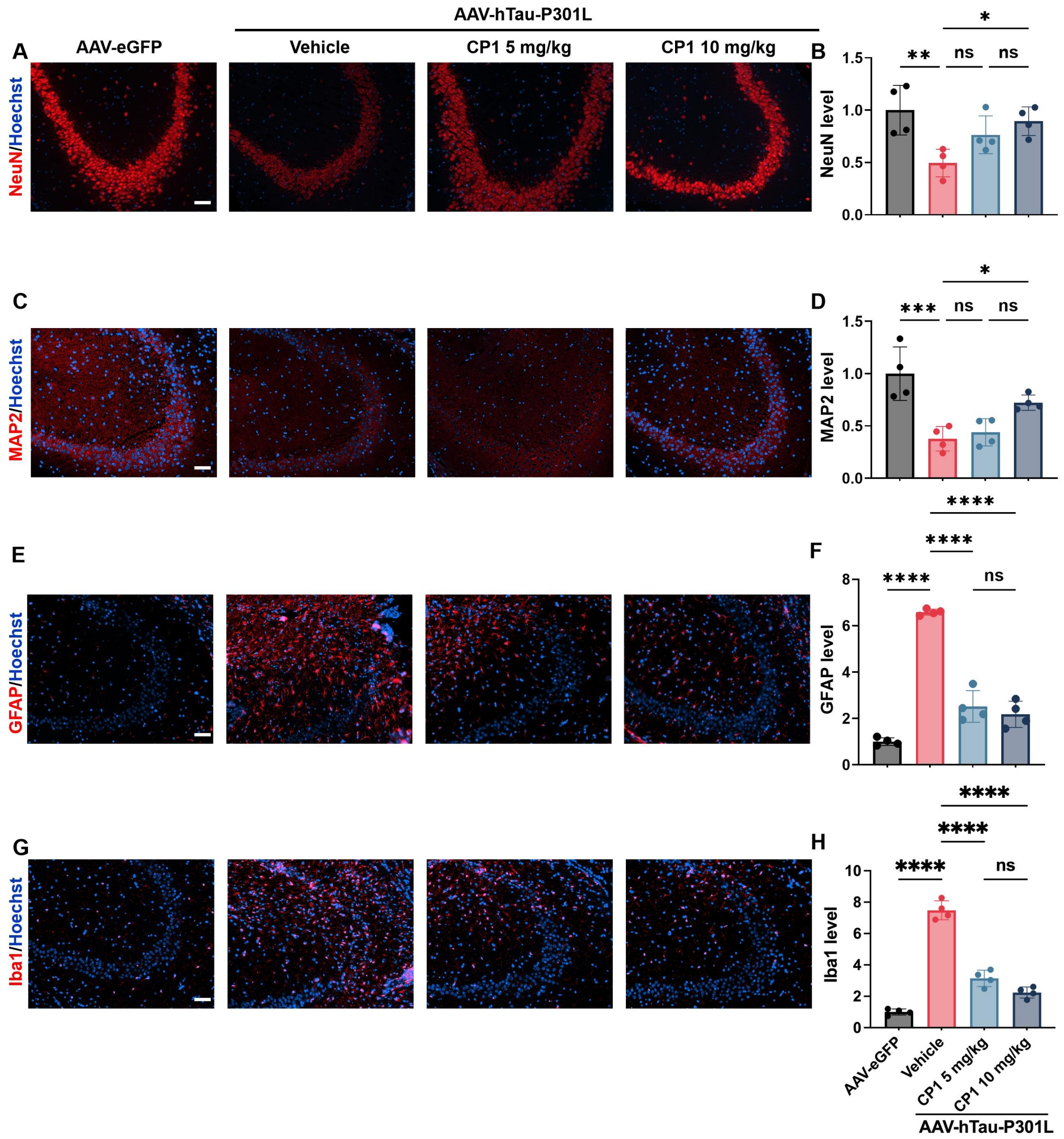

Figure S5. CP1 alleviates neuropathology in male AAV-hTau-P301L mice

(A, B) Representative images of immunofluorescence staining of hippocampal sections with anti-NeuN antibodies (red) and Hoechst 33342 (blue). Scale bar, 50 μm (n = 4 mice per group). *$p < 0.05$, **$p < 0.01$ and ns, not significant, for pairwise comparisons among all groups; one-way ANOVA followed by Tukey's multiple comparisons test. (C, D) Representative images of immunofluorescence staining of MAP2 (red) and Hoechst 33342 staining (blue). Scale bar, 50 μm (n = 4 mice per group). *$p < 0.05$, ***$p < 0.001$ and ns, not significant, for pairwise comparisons among all groups; one-way ANOVA followed by Tukey's multiple comparisons test. (E, F) Representative images of GFAP immunofluorescence staining (red) with Hoechst 33342 (blue). Scale bar, 50 μm (n = 4

mice per group). ****$p< 0.0001$ and ns, not significant, for pairwise comparisons among all groups; one-way ANOVA followed by Tukey's multiple comparisons test. (G, H) Representative images of Iba1 immunofluorescence staining (red) and Hoechst 33342 staining (blue). Scale bar, 50 μm (n = 4 mice per group). ****$p< 0.0001$ and ns, not significant, for pairwise comparisons among all groups; one-way ANOVA followed by Tukey's multiple comparisons test. The quantitative data are presented as the mean ± SD.

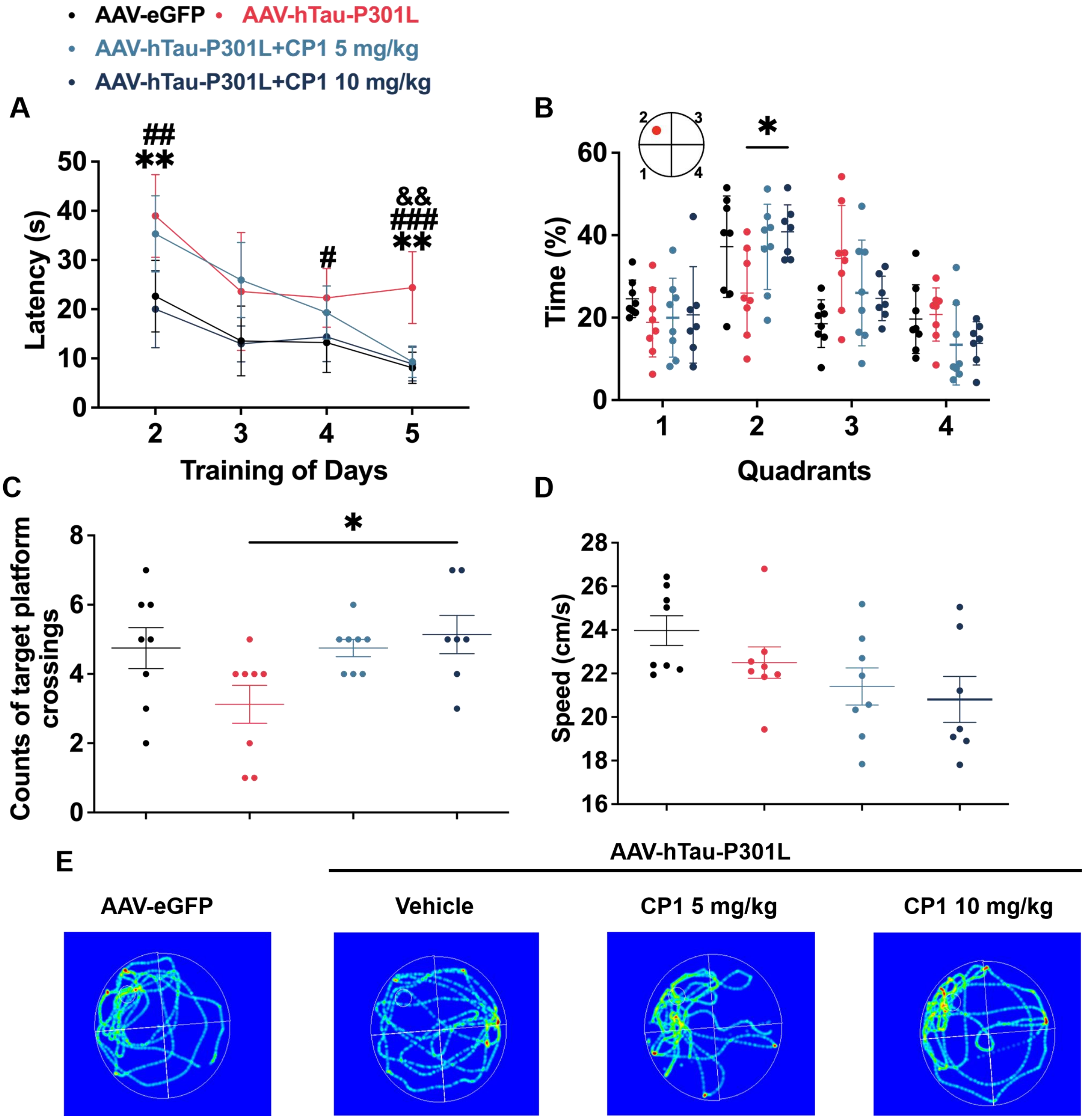

Figure S6. CP1 ameliorates cognitive impairment in male AAV-hTau-P301L mice

(A) MWM training phase assessing spatial learning over consecutive days. (B, C) Probe trial analyses including time spent in the target quadrant (B) and number of platform crossings (C). (D) Average swimming speed during the MWM test. (E) Representative swim trajectories during the probe trial (n = 7-8 mice per group). (A–D) Quantification of behavioral parameters. ***p*< 0.01 indicates significant differences between the AAV-hTau-P301L and AAV-hTau-P301L 10 mg/kg CP1 groups; &&*p* < 0.01 indicates significant differences between the AAV-hTau-P301L and AAV-hTau-P301L 5 mg/kg CP1 groups; #*p* < 0.05, ##*p* < 0.01 and ####*p* < 0.001 indicates significant differences between the AAV-eGFP and AAV-hTau-P301L vehicle groups, data were analyzed using two-way repeated-measures ANOVA followed by Tukey's multiple comparisons test (A); **p* < 0.05

indicates significant differences between the AAV-hTau-P301L and AAV-hTau-P301L 10 mg/kg CP1 groups. One-way ANOVA followed by Tukey's multiple comparisons test unless otherwise indicated (B, C). The quantitative data are presented as the mean ± SD.
